# Macrophages suppress cardiac reprogramming of fibroblasts *in vivo* via IFN-mediated intercellular self-stimulating circuit

**DOI:** 10.1093/procel/pwae013

**Published:** 2024-03-26

**Authors:** Hao Wang, Junbo Yang, Yihong Cai, Yang Zhao

**Affiliations:** State Key Laboratory of Natural and Biomimetic Drugs, Ministry of Education Key Laboratory of Cell Proliferation and Differentiation, Beijing Key Laboratory of Cardiometabolic Molecular Medicine, Institute of Molecular Medicine, College of Future Technology, Peking University, Beijing 100871, China; Peking-Tsinghua Center for Life Science, Academy for Advanced Interdisciplinary Studies, Peking University, Beijing 100871, China; State Key Laboratory of Natural and Biomimetic Drugs, Ministry of Education Key Laboratory of Cell Proliferation and Differentiation, Beijing Key Laboratory of Cardiometabolic Molecular Medicine, Institute of Molecular Medicine, College of Future Technology, Peking University, Beijing 100871, China; State Key Laboratory of Natural and Biomimetic Drugs, Ministry of Education Key Laboratory of Cell Proliferation and Differentiation, Beijing Key Laboratory of Cardiometabolic Molecular Medicine, Institute of Molecular Medicine, College of Future Technology, Peking University, Beijing 100871, China; State Key Laboratory of Natural and Biomimetic Drugs, Ministry of Education Key Laboratory of Cell Proliferation and Differentiation, Beijing Key Laboratory of Cardiometabolic Molecular Medicine, Institute of Molecular Medicine, College of Future Technology, Peking University, Beijing 100871, China; Peking-Tsinghua Center for Life Science, Academy for Advanced Interdisciplinary Studies, Peking University, Beijing 100871, China

**Keywords:** cardiac reprogramming, heart regeneration, microenvironment, *Ifnar1/2*

## Abstract

Direct conversion of cardiac fibroblasts (CFs) to cardiomyocytes (CMs) *in vivo* to regenerate heart tissue is an attractive approach. After myocardial infarction (MI), heart repair proceeds with an inflammation stage initiated by monocytes infiltration of the infarct zone establishing an immune microenvironment. However, whether and how the MI microenvironment influences the reprogramming of CFs remains unclear. Here, we found that in comparison with cardiac fibroblasts (CFs) cultured *in vitro*, CFs that transplanted into infarct region of MI mouse models resisted to cardiac reprogramming. RNA-seq analysis revealed upregulation of interferon (IFN) response genes in transplanted CFs, and subsequent inhibition of the IFN receptors increased reprogramming efficiency *in vivo*. Macrophage-secreted IFN-β was identified as the dominant upstream signaling factor after MI. CFs treated with macrophage-conditioned medium containing IFN-β displayed reduced reprogramming efficiency, while macrophage depletion or blocking the IFN signaling pathway after MI increased reprogramming efficiency *in vivo*. Co-IP, BiFC and Cut-tag assays showed that phosphorylated STAT1 downstream of IFN signaling in CFs could interact with the reprogramming factor GATA4 and inhibit the GATA4 chromatin occupancy in cardiac genes. Furthermore, upregulation of IFN-IFNAR-p-STAT1 signaling could stimulate CFs secretion of CCL2/7/12 chemokines, subsequently recruiting IFN-β-secreting macrophages. Together, these immune cells further activate STAT1 phosphorylation, enhancing CCL2/7/12 secretion and immune cell recruitment, ultimately forming a self-reinforcing positive feedback loop between CFs and macrophages via IFN-IFNAR-p-STAT1 that inhibits cardiac reprogramming *in vivo*. Cumulatively, our findings uncover an intercellular self-stimulating inflammatory circuit as a microenvironmental molecular barrier of *in situ* cardiac reprogramming that needs to be overcome for regenerative medicine applications.

## Introduction

Conditions related to heart failure, such as myocardial infarction (MI) or hypertensive heart disease are among the most physically devastating and globally prevalent health threats (Ambrosy et al., 2014; Molero-Díez et al., 2019). After heart injury, the vast majority of cardiomyocytes (CMs) in the infarct zone undergo irreversible necrosis (Laflamme and Murry, 2005) and the infarct zone is subsequently converted to fibrotic scar tissue populated with cardiac fibroblasts (CFs) (Talman and Ruskoaho, 2016). While several regenerative strategies have been proposed, such as exogenous CM (Kawamura et al., 2012; Qiao et al., 2011; Shiba et al., 2016), induction of endogenous CM proliferation (Mohamed et al., 2018; Nakada et al., 2017; Tao et al., 2016), and tissue patch transplantation (Bejleri et al., 2018; Izadifar et al., 2018; Lin et al., 2019), these therapies cannot eliminate the excessive proliferation of cardiac fibroblasts, which can destroy the myocardial microenvironment and disrupt signal transduction controlling heart contraction.

Direct cardiac reprogramming, especially *in situ*, shows strong potential as a strategy for regenerating cardiomyocytes, as well as for reducing hyperproliferative cardiac fibroblasts. Cardiac fibroblasts can be reprogrammed into cardiomyocytes like cells (iCMs) by overexpressing three transcription factors GMT (*Gata4*, *Mef2c*, and *Tbx5*) (Ieda et al., 2010). However, the low reprogramming efficiency *in vivo*, particularly when using MICFs (cardiac fibroblasts isolated from adult mice with myocardial infarction) as a cell source (Zhao et al., 2021), has hindered its clinical application. Over the past decade, numerous transcription factors (Addis et al., 2013; Garry et al., 2021; Zhou et al., 2015, 2017), small molecule cocktails (Knott et al., 2014; Mohamed et al., 2017; Muraoka et al., 2019; Tao et al., 2023), and miRNA combinations (Jayawardena et al., 2012, 2015; Muraoka et al., 2014) have been shown to improve the efficiency of cardiac reprogramming *in vitro.* Considering the high reprogramming efficiency (~20%) *in vitro*, researchers pursue to convert partially reprogrammed iCMs into fully reprogrammed iCMs with the improved culture condition (Yamakawa et al., 2015). Unfortunately, despite major advances in *in vitro* reprogramming, MICFs conversion efficiency remains remarkably low *in vivo* (~1% in the MI area) (Inagawa et al., 2012; Miyamoto et al., 2018; Song et al., 2012; Tani et al., 2023). Therefore, the problem of excessive differences in cardiac reprogramming efficiency *in vivo* and *in vitro* should be addressed before further improving the maturity of iCMs.

Ineffective virus delivery and the complexity of the *in vivo* microenvironment are some of the major challenges that have limited the effects of reprogramming factors that confer highly potent CFs conversion *in vitro*. In addition, many cardiac reprogramming studies use neonatal mouse CFs (Liu et al., 2016, 2017; Mohamed et al., 2017; Protze et al., 2012; Wang et al., 2015; Zhou et al., 2016) or embryonic fibroblasts as starting cells (Muraoka et al., 2014; Yamakawa et al., 2015; Zhao et al., 2015; Zhou et al., 2017), which have distinctly different transcriptomic profiles from that of *in vivo* target MICFs (Kanisicak et al., 2016). Thus, no *in vitro* culture models have been developed to date that can accurately recapitulate the *in vivo* post-MI microenvironment.

To this end, research has largely focused on improving reprogramming efficiency through the regulation of transcriptional networks, abolishing fibroblast identity, overcoming epigenetic barriers, or screening for additional small molecules (Liu et al., 2021; Vaseghi et al., 2017). However, no studies to date have investigated regenerating CMs from CFs by manipulating the cardiac microenvironment based on the premise that the myocardial infarction microenvironment has complex intercellular communication networks that may impose a strong influence on *in vivo* cardiac reprogramming. After myocardial infarction, heart repair proceeds through several stages, including inflammation, fibroblast proliferation, and remodeling (Frangogiannis, 2012, 2014; Nahrendorf et al., 2010; Prabhu and Frangogiannis, 2016). Inflammation occurs when spleen- or blood-derived monocytes infiltrate the border zone of the injured heart and differentiate into macrophages to clear dead cells (Ma et al., 2018; Prabhu and Frangogiannis, 2016). Leukocytes then secreted many cytokines, such as TGF-β (Dewald et al., 2005; van Amerongen et al., 2007) and IL-10 (Hulsmans et al., 2018), which can stimulate the conversion of cardiac fibroblasts into activated proliferative myofibroblasts, which in turn produce collagen and matrix proteins to remodel the extracellular matrix.

Here, we report the role of the inflammatory microenvironment in impairing CF conversion to CMs *in situ.* By directly comparing the transcriptomic profiles associated with *in vitro* and *in vivo* cardiac reprogramming processes, we identified IFN signaling activity as a major barrier for *in vivo* cardiac reprogramming through IFN-β-IFNAR-p-STAT1 pathway. Genetic knockdown of IFNAR (*Ifnar1* or *Ifnar2*) on MICFs blocks the positive feedback loop of IFN signaling activities between macrophages and MICFs, thus enabling the efficient MICFs reprogramming into cardiomyocytes in mice with MI *in vivo*.

## Results

### Silencing *Ifnar1* or *Ifnar2* in cardiac fibroblasts decreased IFN pathway activity and increased cardiac reprogramming efficiency *in vitro*

**Figure 1.**
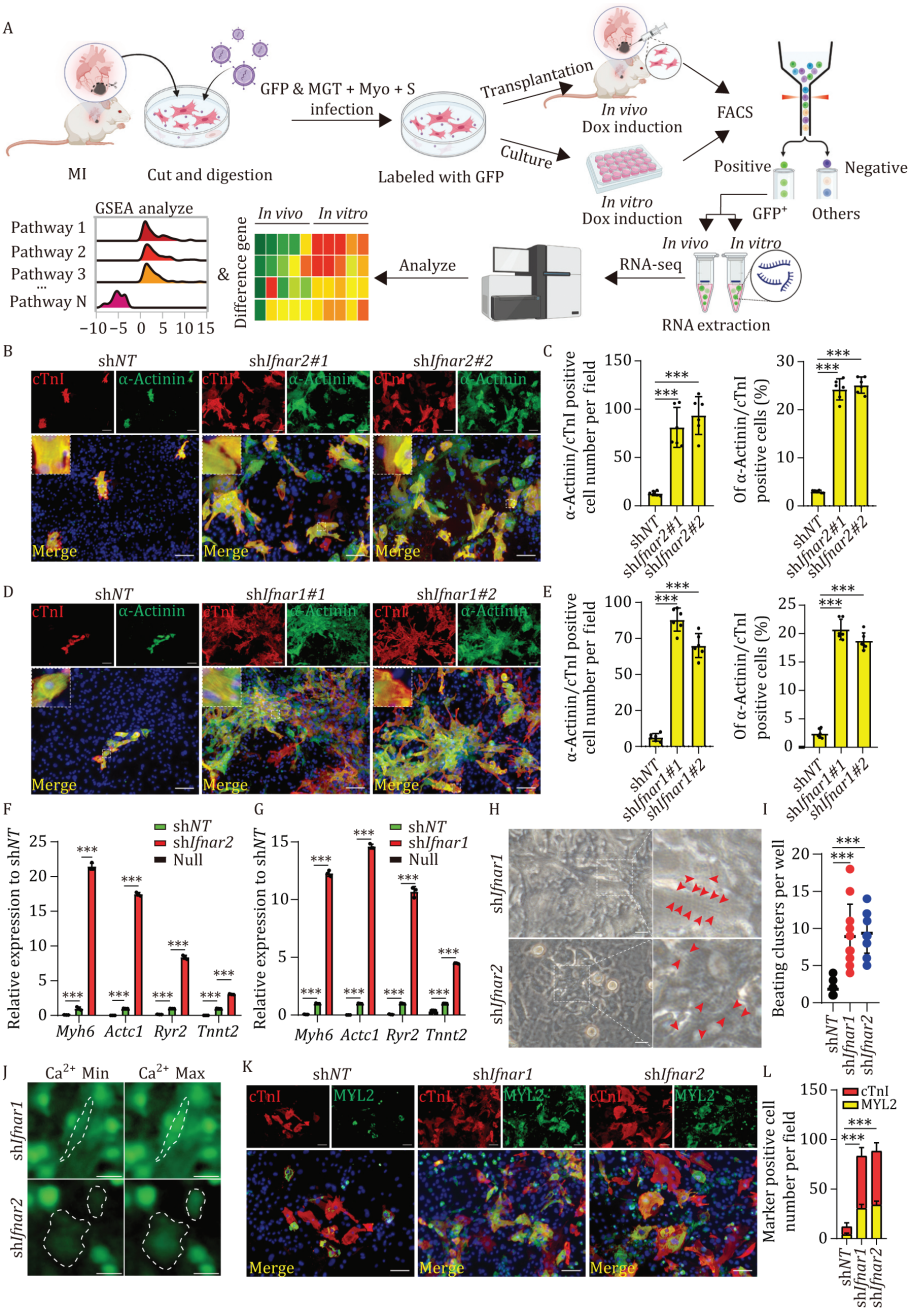
Silencing *Ifnar1* or *Ifnar2* reduced IFN pathway response and increased cardiac reprogramming efficiency in MICFs. (A) Schematic diagram of the reprogrammable cell transplantation system to compare transcriptional profiles from *in vitro* and *in vivo* samples. Of note, all reprogramming factor constructs and eGFP construct were tetracycline inducible (Tet-On system). (B–E) Representative immunofluorescence (IF) images for α-actinin and cTnI on MICFs infected with MGT and sh*Ifnar1*/*2* or sh*NT* (B and D), with quantification of the absolute number and the percentage in (C and E). High magnification views in insets show the sarcomeric organization, *n* = 6. (F and G) Relative mRNA expression levels in iCMs after 4 weeks of *Ifnar1* or *Ifnar2* KD compared with sh*NT* control. Null: MICFs without virus infection, *n* = 3. (H) Representative images of iCMs exhibiting well-formed sarcomere structures with 4 weeks induction. (I) Quantification of beating iCMs in one well of a 24-well plate after 4 weeks induction, *n* = 4–9, corresponding to Movie S1 and Movie S2. (J) Representative images of iCMs exhibiting calcium transient after 4 weeks induction, corresponding to Movie S3 and Movie S4. (K and L) Representative IF images for MYL2 and cTnI on MICFs infected with MGT and sh*Ifnar1*/*2* or sh*NT* (K), with quantification of the absolute number (L), *n* = 3. All data are presented as the means ± SD. ns, not significant, *P < 0.05, **P < 0.01, ***P < 0.001. The One-way ANOVA was used to determine the significance of differences between two groups. Scale bars, 100 μm (H = 20 μm, J = 25 μm). See also Figs. S1 and S2.

In order to investigate the *in vivo* suppressors of CFs reprogramming, we established a transplantation system that enables the comparison of the same cells expressing a combination of reprogramming factors between *in vivo* and *in vitro* conditions, to preclude potential differences in gene delivery efficiency (Fig. 1A). Briefly, we digested *in vitro* cultured MICFs (cardiac fibroblasts isolated from adult mice with myocardial infarction) infected with lentiviruses tetracycline-inducible expressing MGT (polycistronic construct including *Mef2c*, *Gata4*, and *Tbx5*), Myo + S (separates virus driving expression of *Myocd* and *Sall4*) (Zhao et al., 2021), and eGFP, respectively, and transplanted them into hearts of mice with surgically induced MI (MI hearts hereafter). A portion of these infected MICFs were retained for concurrent *in vitro* culture with the addition of doxycycline (Dox). On Days 7 and 14 after transplantation and Dox administration, we used FACS to isolate eGFP^+^ transplanted MICFs from the hearts of MI mice and compare them with FACS-isolated eGFP^+^ MICFs with sustained *in vitro* culture (Fig. 1A).

RNA-seq analysis comparing these eGFP^+^ cells from MI mice or *in vitro* cultures showed that the expression of cardiomyocyte-related genes (including *Tnni3*, *Actc1*, *Tnnt2*, *Myh6*, and *Ryr2*) was significantly suppressed in the *in vivo* samples, while fibroblast-related genes (including *Col1a1*, *Col3a1*, *Fn1,* and *Spp1)* were markedly upregulated compared with the *in vitro* samples (Fig. S1A). We also noted that genes involved in the IFN-α/β and IFN-γ pathways were expressed at markedly higher levels in the *in vivo* samples (Fig. S1B and S1C). Further analysis of differentially expressed genes (DEGs) revealed that most interferon-stimulated genes (ISGs), including *Mx1*, *Ifit1*/*3*, *Usp18*, *Isg15*/*20*, *Oas1*/*2*/*3* were significantly upregulated in the *in vivo* samples compared with their expression in the *in vitro* samples (Fig. S1D and S1E). These results suggested the possibility that IFN-related signaling pathways might be involved in resisting MICFs reprogramming *in vivo*.

To explore the potential role of IFN signaling in suppressing cardiac reprogramming, we employed short hairpin RNAs (shRNA) to specifically knockdown (KD) the interferon receptors (IFNAR) genes *Ifnar1* or *Ifnar2* (IFNAR were formed by two chains encoded by *Ifnar1* and *Ifnar2*) to reduce the response of IFN-α/β in MICFs isolated from mice at Day 6 post myocardial infarction (MI D6), with the expression of MGT or MGTMyoS (Fig. S2A). Immunofluorescence (IF) staining of cultured MICFs expressing MGT or MGTMyoS *in vitro* indicated that KD of either *Ifnar1* or *Ifnar2* led to increased reprogramming efficiency compared with the non-targeted shRNA controls (sh*NT)* (Fig. S2B and S2C). MGT was used as the basic reprogramming factor combination in all subsequent experiments for more convenience, and CFs to CMs reprogramming efficiency was quantified by monitoring co-expression of two CMs markers, α-actinin and cardiac troponin I (cTnI, also known as *Tnni3*). Image-based statistical analysis of cultured induced cardiomyocyte-like cells (iCMs) using the high-content analysis platform (cell discovery 7, CD7) and flow cytometry showed that suppression of *Ifnar1* or *Ifnar2* resulted in an approximate 10-fold increase in the percentage of α-actinin ^+^cTnI^+^ double-positive cells compared with that in the sh*NT* controls (Figs. 1B–E and S2D–F), indicating that the transduction of IFN signaling pathway possibly hindered the cardiac reprogramming of MICFs.

We then examined whether *Ifnar1* or *Ifnar2* KD also affected the maturation and function of iCMs using qPCR-based assessment of sarcomere and ion channel marker expression, including *Myh6*, *Actc1*, *Tnnt2*, and *Ryr2*. In agreement with our IF results, all of these CMs markers were expressed at ~5–20-fold higher levels in *Ifnar1* or *Ifnar2* KD group *in vitro* compared with that in sh*NT* control group after 4 weeks (Fig. 1F and 1G). Further observation by brightfield microscopy revealed the presence of sarcomere structures (Fig. 1H), as well as numerous spontaneous beating iCMs in *Ifnar1* or *Ifnar2* KD group after 4 weeks (Fig. 1I; Movies S1 and S2), but few in the control group. Fluorescent probes for calcium flux confirmed the periodic calcium oscillation in spontaneously beating iCMs (Fig. 1J; Movies S3 and S4). In addition, approximately 50% of iCMs with *Ifnar1* or *Ifnar2* KD also expressed MYL2, a marker for ventricular cardiomyocytes (Fig. 1K and 1L). These results suggested that silencing *Ifnar1* or *Ifnar2* resulted in a higher ratio of cells containing sarcomere structures and a larger population of iCMs with enhanced functionality *in vitro*. Taken together, these results showed that the efficiency of iCMs reprogramming could be enhanced by suppressing *Ifnar1* or *Ifnar2*, and implied that this reprogramming system was robust for generating functional iCMs from MICFs in adult mice.

### Suppression of *Ifnar2* improves the efficiency of cardiac reprogramming *in vivo*

**Figure 2.**
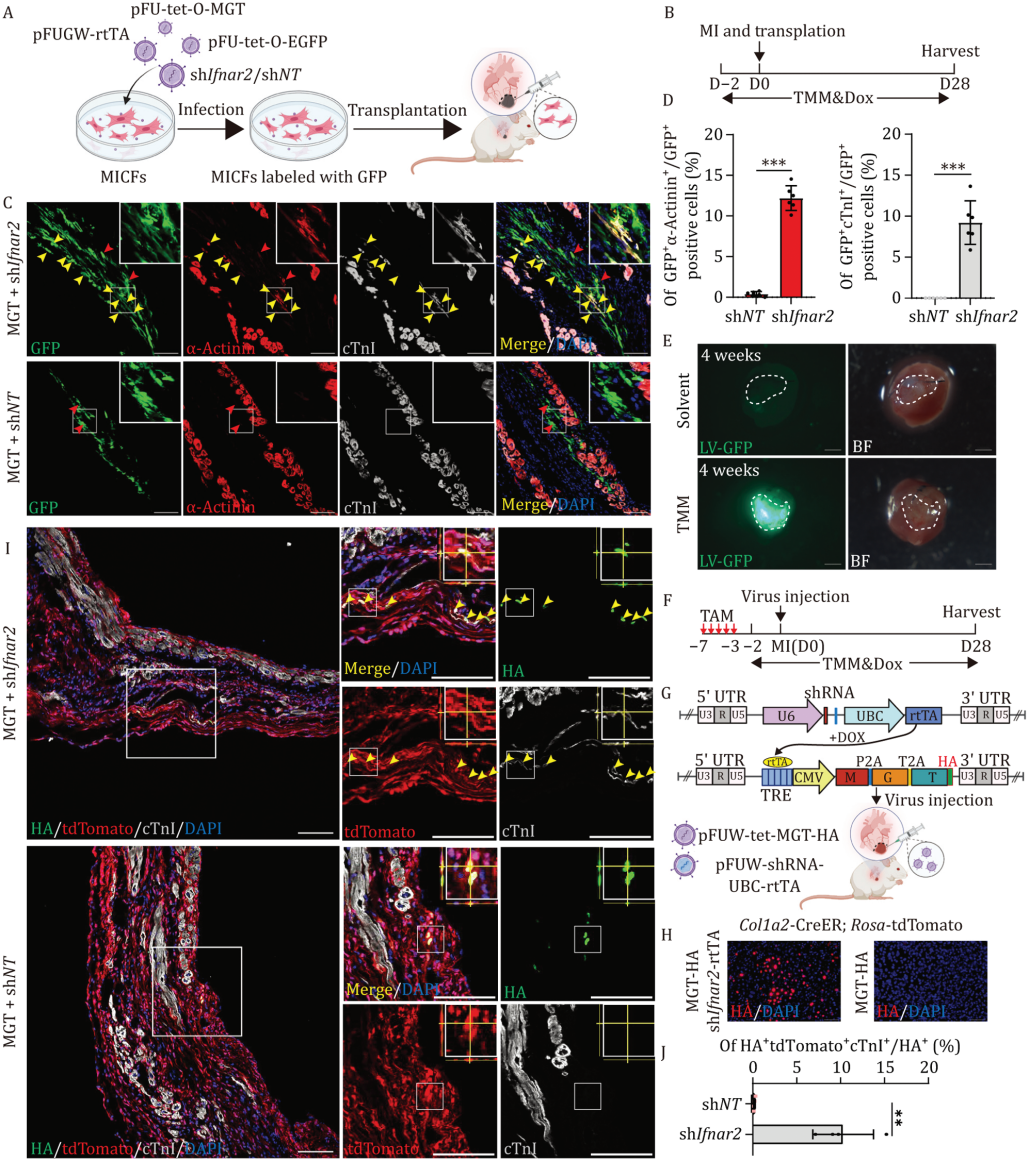
Downregulation of *Ifnar2* enhances cardiac reprogramming efficiency *in vivo*. (A and B) Schematic diagram of MICFs infected with MGT, eGFP and sh*Ifnar2* or sh*NT* transplanted into C57BL/6 mouse hearts (A). Experimental workflow of MICFs transplantation, time window for immunosuppressant cocktail (TMM) and Dox administration (B). (C and D) Representative IF images for α-actinin, cTnI, and GFP after transplantation of MICFs expressing MGT and sh*Ifnar2 or* sh*NT* into MI hearts 4 weeks (C), with quantification of the percentage in (D). Red arrow indicates α-actinin^+^cTnI^−^ iCMs, Yellow arrow indicates α-actinin^+^cTnI^+^ iCMs, *n* = 6. (E) eGFP^+^ cells were eliminated by immune cells after 4 weeks in C57BL/6 hearts, whereas it remained up to 4weeks in C57BL/6 mice injected with TMM per day, *n* = 4. (F) Experimental workflow of virus injection, timeline for immunosuppressant cocktail (TMM), Dox and tamoxifen (TAM) administration. (G) Schematic diagram depicted optimized lentivectors allowed co-infection with MGT-HA (HA fusion expression with TBX5) and sh*Ifnar2* or sh*NT* based on Tet-on system. (H) Representative IF images for HA on MICFs infected with MGT-HA and sh*Ifnar2-rtTA* or MGT-HA, *n* = 3. (I and J) Representative IF images for tdTomato, HA and cTnI after injection of LV-MGT-HA and LV-shRNA-rtTA into *Col1a2*^CreER^/*R26*-tdTomato MI hearts 4 weeks (I), high magnification views in insets show z stack for HA tdTomato and cTnI positive iCMs, with quantification of the percentage in (J), *n* = 4. All data are presented as the means ± SD. The unpaired *t*-test was used to determine the significance of differences between two groups. ns, not significant, *P < 0.05, **P < 0.01, ***P < 0.001, Scale bars, 100 μm (E = 1000 μm and high magnification views in G = 50 μm). See also Fig. S3.

In light of the above results showing efficient reprogramming *in vitro*, we sought to determine whether downregulation of *Ifnar1* or *Ifnar2* could also improve reprogramming efficiency *in vivo*. Since the reprogramming efficiency with *Ifnar2* KD was slightly higher than that of *Ifnar1* KD *in vitro*, we employed sh*Ifnar2* for the following *in vivo* reprogramming experiments. First, MICFs co-infected with lentiviruses expressing eGFP, MGT, and sh*Ifnar2* (or the sh*NT* control construct) were transplanted into the hearts of mice during surgically-induced MI (Fig. 2A). The hearts were harvested at 4 weeks after transplantation and Dox induction (Fig. 2B). IF staining of cryo-sections showed that approximately ~12.2% eGFP^+^ cells in the sh*Ifnar2* group were α-actinin^+^, ~9.2% were cTnI^+^ and ~8.2% were cTnI^+^α-actinin^+^, whereas rare α-actinin^+^ iCMs (~0.4%) were observed in the control group after 4 weeks (Fig. 2C and 2D). In addition, ~6.5% eGFP^+^ cells were cTnI^+^ MYL2^+^ and ~75% cTnI^+^eGFP^+^ cells expressed MYL2 (Fig. S3A and S3B), indicating most iCMs might be ventricular cardiomyocytes-like cells. Of note, α-actinin^+^eGFP^+^ cells were more abundant among the transplanted cells than cTnI^+^eGFP^+^ cells in hearts of *Ifnar2* KD mice, which was consistent with the *in vitro* results. It indicated that *Ifnar2* KD could reverse the IFN inhibition effect in transplanted MICFs, enabling efficient cardiac reprogramming *in vivo*.

We then investigated whether *Ifnar2* silencing affected resident MICFs reprogramming into iCMs *in situ* through direct virus delivery. To determine the origin of newly generated iCMs *in vivo*, we applied a *Col1a2*^CreER^/*R26*-tdTomato lineage-tracing system to track the cell fate of MICFs (Fig. S3C). The *Col1a2*^CreER^/*R26*-tdTomato mice were treated with tamoxifen (TAM) for 5 days to label cells expressing *Col1a2*^CreER^. Coronary artery ligation was performed on 7 days after the first tamoxifen treatment (Dirkx et al., 2013); Dox was administered 2 days prior to virus delivery and continued until harvest. To evaluate the delivery efficiency of exogenous genes to *Col1a2*^CreER^-labeled cells, we injected two lentiviruses, pTRE-GFP and pUBC-rtTA, into the hearts of *Col1a2*^CreER^/*R26*-tdTomato mice. However, both the transplantation and virus injection processes activated an endogenous immune response in mice, leading to the elimination of virus-infected cells, as previously reported by Ieda group (Inagawa et al., 2012) (Fig. 2E). To address this issue, an immunosuppressant cocktail that consists of tacrolimus, mycophenolate mofetil, and methylprednisolone (TMM) was administered to attenuate immune responses after virus injection (Fig. 2E). At 4 weeks post virus injection and TMM administration (Fig. 2F), ~80.0% eGFP^+^ cells were tdTomato^+^ in the injured areas, which indicated a high efficiency of exogenous gene delivery to target cells (Fig. S3D and S3E).

Considering that the co-infection efficiency is low *in vivo*, we generated a tandem lentiviral vector containing both pUBC-rtTA and pU6-sh*Ifnar2* or pU6-sh*NT* instead of two independent vectors (Fig. 2G). The reprogramming efficiency of tandem vector and independent vector was comparable (Fig. S3F and S3G). After that we directly injected lentiviruses pTRE-MGT-HA and pUBC-rtTA-U6-sh*Ifnar2* or the pUBC-rtTA-U6-sh*NT* control, into the hearts of *Col1a2*^CreER^/*R26*-tdTomato mice (Fig. 2G). It should be noted that the HA tag expressed by pTRE-MGT-HA enabled observation of cells that were co-infected with both the reprogramming factors and the shRNA (Fig. 2H). Four weeks after virus injection and TMM administration, IF staining of cryo-sections showed that ~10.3% of the HA^+^ tdTomato^+^ cells expressed cTnI after delivery of MGT + sh*Ifnar2*, while rare (~0.2%) HA^+^ tdTomato^+^ cells expressed cTnI in the control group (Fig. 2I, 2J). Adjacent transverse sections stained for α-actinin showed that up to ~89.6% HA^+^ tdTomato^+^ cTnI ^+^cells also expressed α-actinin (Fig. S3H). Three-dimensional analyses of cryo-sections and adjacent transverse sections confirmed the presence of HA^+^tdTomato^+^ and cTnI^+^ or tdTomato^+^ and α-actinin^+^ cells with *Ifnar2* KD (Fig. S3I).

Since cell fusion events are critical for verifying bona fide cardiac regeneration, we investigated whether iCMs originated through cardiac reprogramming or fusion with resident CMs. We generated *Tcf2*1-CreER/mTmG/mice (Fig. S3J) that constitutively expressed membrane-bound tdTomato (mtdTomato) from the *Rosa26* locus. Subsequent CreER-mediated recombination driven by *Tcf21* in MICFs, resulted in deletion of mtdTomato and instead permanent expression of a membrane-targeted eGFP (mGFP). Thus, direct cardiac reprogramming from CFs in these mice was indicated by the presence of mGFP signal alone, whereas cells generated through CF-CM fusion co-expressed both mtdTomato and mGFP (Fig. S3J). After 4 weeks TMM administration and injection of pTRE-MGT-HA and pUBC-rtTA-U6-sh*Ifnar2* or pUBC-rtTA-U6-sh*NT* lentiviruses, IF staining of cryo-sections showed that ~5.2% of the HA^+^mGFP^+^ cells expressed cTnI after delivery of the MGT + sh*Ifnar2* lentiviruses, while rare ~0.15% HA^+^mGFP^+^ cells expressed cTnI in the MGT + sh*NT* control group (Fig. S3K and S3L). Staining for α-actinin in adjacent transverse sections showed that HA^+^mGFP^+^cTnI^+^ cells also expressed α-actinin (Fig. S3M and S3N). Moreover, in the MGT + sh*Ifnar2* group, a small number of mGFP^+^mtdTomato^−^ and α-actinin^+^ cells exhibited well-organized sarcomere structure (Fig. S3O). These results thus indicated that transduction of MGT + sh*Ifnar2* could reprogram resident CFs into iCMs in infarcted heart tissue of mice.

### *In vivo* delivery of MGT + sh*Ifnar2* improves cardiac function and ameliorates fibrosis after myocardial infarction

**Figure 3.**
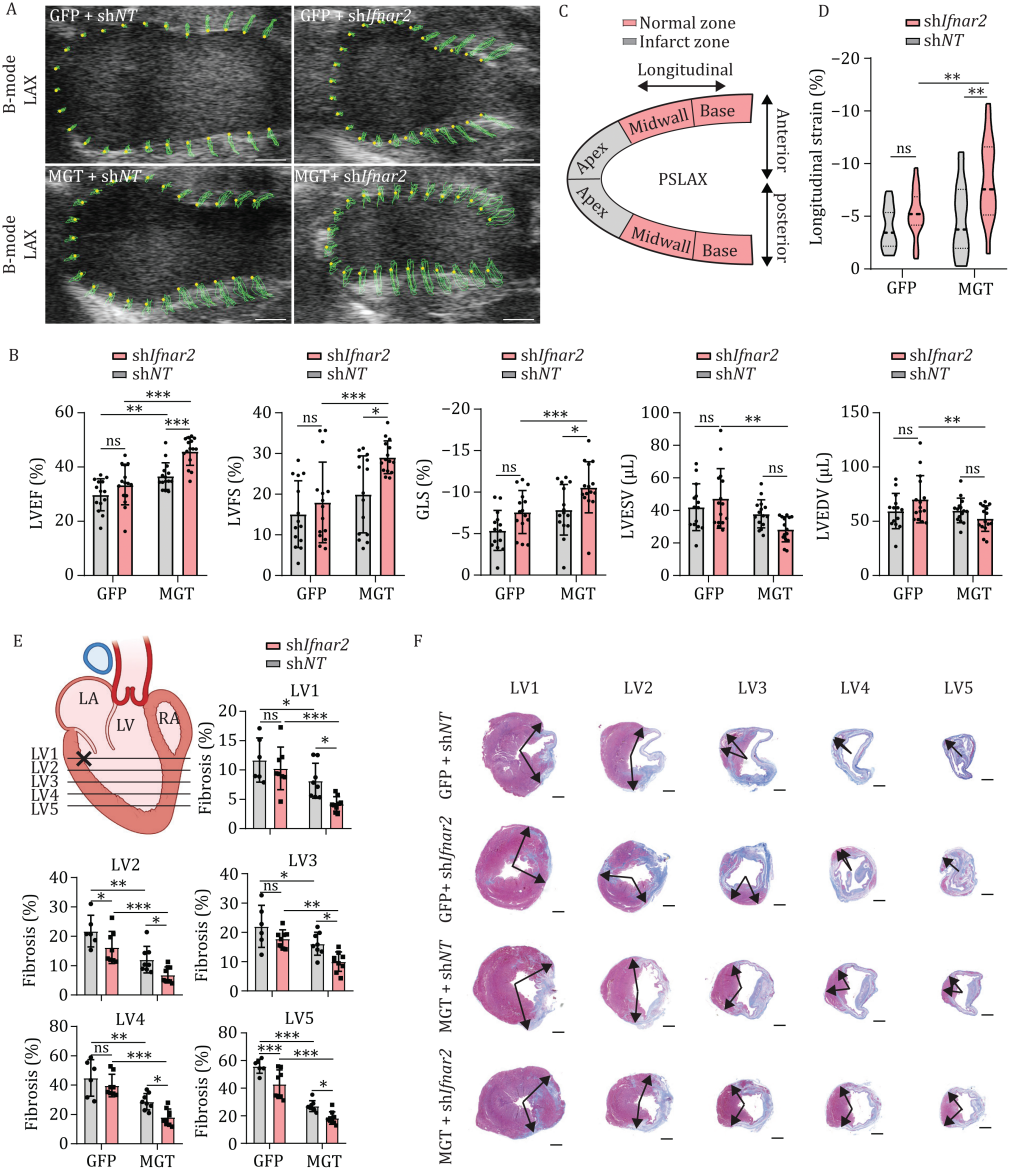
Transduction of MGT + sh*Ifnar2* improves cardiac function and reduces fibrosis after MI. (A and B) B-mode of echocardiographic parameters for the C57BL/6 mice injected with GFP + sh*NT*, GFP + sh*Ifnar2*, MGT + sh*NT*, or MGT + sh*Ifnar2* lentiviruses at 28 after MI (A). Quantification of ejection fraction (EF), fractional shortening (FS), global longitudinal strain (GLS), end-systolic volume (ESV) and end-diastolic volume (EDV) for the mice injected with GFP + sh*NT*, GFP + sh*Ifnar2*, MGT + sh*NT* or MGT + sh*Ifnar2* lentiviruses at 28 after MI (B), *n* = 14–15. (C) Schematic diagram showing parasternal long axis (PSLAX) view of the heart. (D) Quantification of the longitudinal peak strains from the posterior apex for the mice injected with GFP + sh*NT*, GFP + sh*Ifnar2*, MGT + sh*NT* or MGT + sh*Ifnar2* lentiviruses at Day 28 after MI, results were presented as violin plots, *n* = 14–15. (E and F) Comparison of fibrosis areas among the GFP + sh*NT*, GFP + sh*Ifnar2*, MGT + sh*NT* or MGT + sh*Ifnar2* lentiviruses injection groups 4 weeks after MI. Fibrosis was evaluated at five levels (LV1–LV5) (E) by Mason staining. Representative histology images (F) and quantitative analyses for the fibrotic area at each level are shown in (E), *n* = 6–9. All data are presented as the means ± SD. The two-way ANOVA was used to determine the significance of differences between two groups. ns, not significant, *P < 0.05, **P < 0.01, ***P < 0.001. See also Fig. S4. Scale bars, 1 mm.

Given the above results showing that suppression of *Ifnar2* can increase reprogramming efficiency of resident MICFs *in vivo*, we next asked whether gene transduction of MGT + sh*Ifnar2* could improve cardiac function after MI. To this end, echocardiography was used to assess cardiac function in C57BL/6J mice injected with GFP + sh*Ifnar2*, GFP + sh*NT*, MGT + sh*Ifnar2*, or MGT + sh*NT* lentiviruses in a blinded fashion at 4-weeks post-MI. Among them, the MGT + sh*Ifnar2* group exhibited the greatest functional improvements compared to the MGT + sh*NT* controls in left ventricle ejection fraction (LVEF; 45.8% vs.36.7%), left ventricle fractional shortening (LVFS; 29.1% vs.19.9%), and global longitudinal strain (GLS; −10.6% vs. −7.9%) (Fig. 3A and 3B).

To investigate functional recovery in finer detail, we divided the left ventricle (LV) into six segments along the parasternal long axis (PSLAX) view (Fig. 3C), since segmental analysis from base to apex of anterior and posterior could capture subtle changes that could be overlooked in parameters such as longitudinal segment. Measurement of longitudinal segment strains from the GFP + sh*NT*, GFP + sh*Ifnar2*, MGT + sh*NT*, and MGT + sh*Ifnar2* lentiviruses injected groups showed no differences in base or mid-ventricle strains across groups, irrespective of location in the posterior or anterior walls (Fig. S4A). However, echocardiograms indicated that longitudinal segment strain was significantly stronger in the posterior apex (infarcted zone) of MGT + sh*Ifnar2* group MI mice compared to MGT + sh*NT* controls at 4 weeks (−7.9% vs. −5.4%; Fig. 3D), suggesting that recovery of cardiac function in the infarcted area was greater in the MGT + sh*Ifnar2* group.

We next conducted histological analyses by Mason staining to quantify scar size after 4 weeks of lentiviruses injection. Blinded quantification of serial sections sampled from five layers of the left ventricle (LV1–LV5; Fig. 3E) revealed that scar area was significantly reduced in MGT + sh*NT* mice compared with the GFP + sh*NT* control mice (Fig. 3E and 3F). Moreover, the MGT + sh*Ifnar2* group had significantly less scar area than the MGT + sh*NT* group, suggesting that the MGT + sh*Ifnar2* treatment resulted in greater reduction of fibrosis in infarcted mouse hearts than either MGT treatment or *Ifnar2* silencing alone (Fig. 3E and 3F). Taken together, these results demonstrated that *in vivo* delivery of MGT + sh*Ifnar2* could improve cardiac function and reduce fibrosis after MI.

### IFN-β secreted by macrophages hinders CFs reprogramming *in vivo*

**Figure 4.**
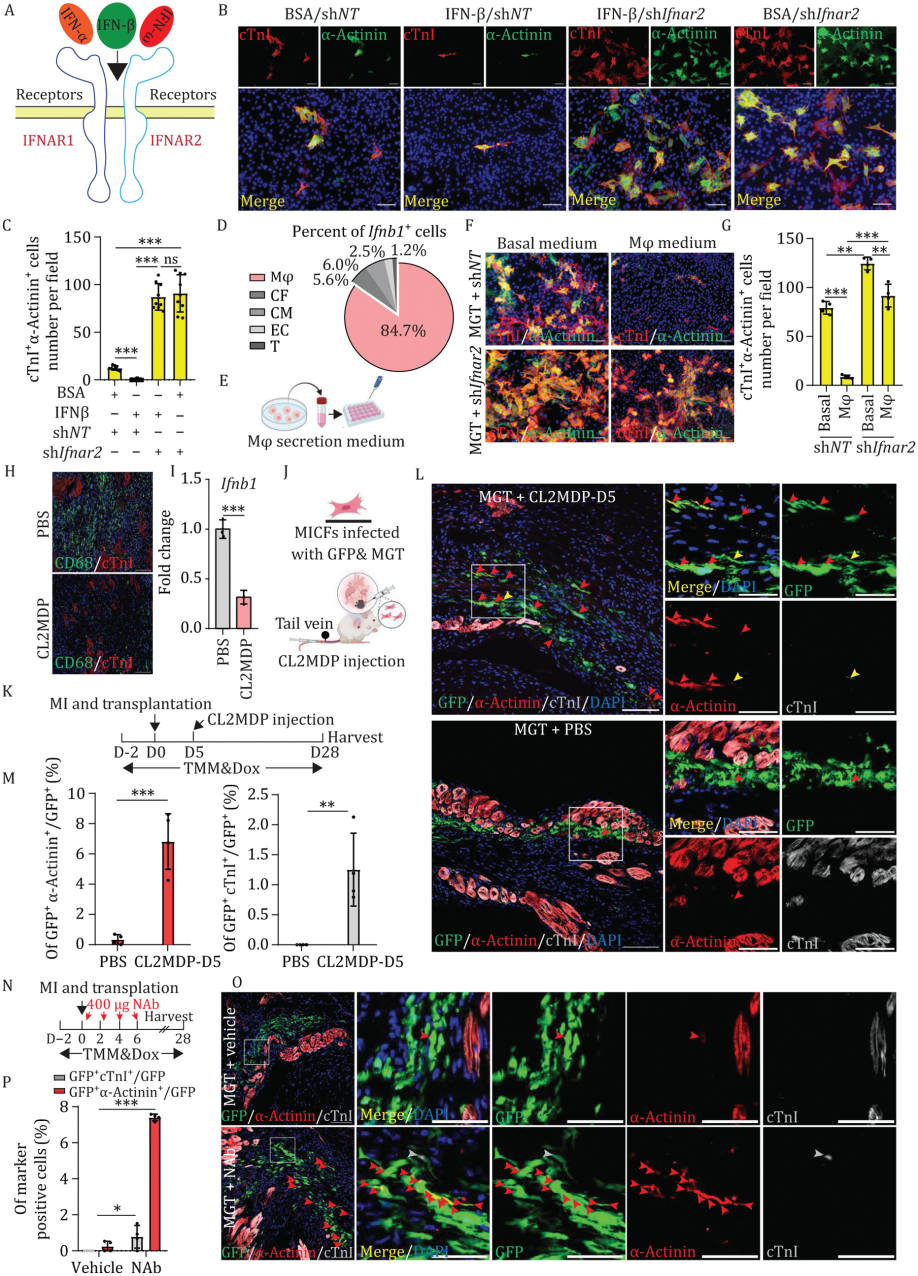
Macrophage hinder MICFs reprogramming *in vivo* by secretion IFN-β. (A) Schematic diagram of type I IFN-IFNAR axis. (B and C) Representative IF images for α-actinin and cTnI on MGT + sh*Ifnar2* or sh*NT* transduced MICFs treated with 25 IU/mL IFN-β or BSA (B), with quantification of the absolute number in (C), *n* = 9. (D) Pie chart showing percentage distribution of *Ifnb1* expression in different cell types in mouse hearts with injury. Mφ: macrophages, CF: cardiac fibroblasts, CM: cardiomyocytes, EC: endothelial cells, T: T cells. (E) Schematic diagram of bone marrow-derived macrophages-conditioned medium. (F and G) Representative IF images for α-actinin and cTnI on MGT + sh*Ifnar2* or sh*NT* transduced purified MICFs treated with conditional medium or basal medium (F), with quantification of the absolute number in (G), *n* = 4. (H) IF staining of CD68 after treatment of CL2MDP or PBS 2 days in mouse hearts, *n* = 4. (I) RT-PCR analyses for *Ifnb1* gene expression at 2 days after treatment of CL2MDP or PBS in mouse hearts, *n* = 3. (J and K) Schematic diagram (J) and experimental timeline (K) of the C56BL/6 mice treated with CL2MDP, TMM and Dox. (L and M) Representative IF images for α-actinin, cTnI, and GFP on transplantation of MGT-transduced MICFs treated with CL2MDP or PBS into MI hearts 4 weeks (L), with quantification of the percentage in (M). Red arrow indicates α-actinin^+^cTnI^−^ iCMs; Grey arrow indicates α-actinin^−^cTnI^+^ iCMs; Yellow arrow indicates α-actinin^+^cTnI^+^ iCMs, *n* = 4. (N) Schematic diagram showing the timeline of IFNAR-neutralizing antibody injection and administration of TMM and Dox. (O and P) Representative IF images for α-actinin, cTnI, and GFP on transplantation of MGT-transduced MICFs treated with IFNAR-NAb into MI hearts (O), with quantification of the percentage in (P). Red arrow indicates α-actinin^+^cTnI^−^ iCMs; Grey arrow indicates α-actinin^−^cTnI^+^ iCMs, *n* = 4. All data are presented as the means ± SD. The two-way ANOVA (C and G) or unpaired *t*-test (I, M, and P) was used to determine the significance of differences between two groups. ns, not significant, *P < 0.05, **P < 0.01, ***P < 0.001, Scale bars, 100 μm (high magnification views in K = 50 μm). See also Figs. S5 and S6.

After confirming that silencing of IFNAR receptors can increase reprogramming efficiency *in vivo* and *in vitro,* we next investigated cellular and molecular mechanisms through which IFN signaling pathway factors could suppress cardiac reprogramming and sought to identify the cells that secreted these factors. Previous studies have reported that IFNAR function as type I interferon receptors, recognizing IFN-α, IFN-β, IFNω, etc., which are known to activate MICFs (de Weerd and Nguyen, 2012) (Fig. 4A). Analysis of publicly available single cell RNA-seq (scRNA-seq) data (GSE120064) revealed that only *Ifnb1* was highly expressed after cardiac injury, while other type I interferon-related genes were expressed at lower levels or were undetectable (Fig. S5A and S5B). We therefore speculated that IFN-β may play a function in blocking MICFs conversion after cardiac injury. To explore this possibility, we induced myocardial infarction in mice and harvested the infarcted area of hearts at 3-, 5-, 14-, 21-, and 28-days post-MI. Quantification by qPCR indicated that IFN-β expression was significantly upregulated beginning at MI D3 and remained high until MI D28 (Fig. S5C), suggesting that high expression of IFN-β could potentially hinder MICFs reprogramming after myocardial infarction.

To examine the effects of IFN-β on CFs reprogramming, we treated MGT + sh*NT*/sh*Ifnar2* infected cells with IFN-β or BSA *in vitro*. After 4 weeks of induction and IFN-β treatment, IF staining of MGT + sh*NT* group showed that significantly fewer α-actinin^+^ cTnI^+^ iCMs were present with IFN-β treatment (Fig. 4B and 4C) suggesting that IFN-β served as an exogenous suppressor of cardiac reprogramming. Besides, the inhibitory effect of IFN-β was abolished in cells with *Ifnar2* KD (Fig. 4B and 4C), indicating that IFN-β exerted reprogramming inhibitory effects on MICFs through IFNAR.

To identify cells that secreted IFN-β and to understand the process of cell interaction in the myocardial infarction microenvironment, we then re-explored publicly scRNA-seq data to analyze IFN-β expression in different cell types. This scRNA-seq analysis revealed that macrophages accounted for ~84.7% of all IFN-β expressing cells in heart samples of injured mice (Figs. 4D and S5D), consistent with other studies (King et al., 2017). These results suggested that macrophages were likely responsible for the majority of IFN-β secretion, and consequently might suppress CFs conversion to CMs. Since MICFs isolated from MI D6 hearts comprised 80.9% cardiac fibroblasts, 17.0% macrophages, and 1.1% endothelial cells (Fig. S5E and S5F), we next examined whether depleting macrophages could increase CFs reprogramming efficiency. To this end, we employed MACS CD45-magnetic beads to eliminate macrophages resident in MICFs (Fig. S5G). After 4 weeks of transduction, IF staining showed that MICFs purification (i.e., macrophage depletion) significantly increased the number of cTnI^+^α-actinin^+^ cells by ~7.2-fold, a comparable level to *Ifnar2* KD, and their combination did not further increase iCMs induction efficiency, indicating that macrophages played a major role in suppressing CFs-CMs conversion, probably through IFN signaling (Fig. S5H).

Moreover, we cultured bone marrow-derived macrophages (BMDMs) obtained from naïve wild-type mice *in vitro* (Fig. S6A) and collected culture supernatants after 5 days (Fig. 4E). We then added this conditional medium to the reprogramming medium for culturing of purified MICFs infected with MGT + *shNT* or MGT + *shIfnar2.* After 4 weeks, IF analysis showed that the number of cTnI^+^α-actinin^+^ cells in MGT + sh*NT* MICFs significantly declined by ~9.1-fold in conditional medium compared with that in basal control cultures, whereas the cells treated with MGT + sh*Ifnar2* showed a slight decrease (~1.4-fold) in double-positive cells (Fig. 4F and 4G), indicating the inhibitory effect of macrophage was decreased after KD *Ifnar2*.

To further confirm the role of macrophages in hindering CFs reprogramming to CMs via IFN-β secretion, we generated *Ifnb1* knockout (KO) BMDMs (Fig. S6B) and collected culture supernatants after 5 days. We then added this KO-conditioned medium or conditioned medium from WT BMDMs to the basal reprogramming medium for culturing of purified MICFs treated with MGT + sh*NT*/sh*Ifnar2*. After 4 weeks of transduction, IF staining indicated that the number of cTnI^+^α-actinin^+^ cells significantly declined by ~8.8-fold in the MGT + sh*NT* group treated with conditioned medium compared with the basal medium-only controls, whereas cells treated with KO-conditioned medium showed only a negligible decrease in cTnI^+^α-actinin^+^ cells compared with the basal medium controls (Fig. S6C and S6D), indicating that the inhibitory effect of macrophage was decreased by *Ifnb1* KO. By contrast, the number of cTnI^+^α-actinin^+^ cells showed a slight decrease in MGT + sh*Ifnar2* group treated with either conditioned medium or KO-conditioned medium compared with the basal medium controls (Fig. S6C and S6D). Taken together, these results suggested that IFN-β secreted by macrophages could inhibit CFs to CMs conversion *in vitro*.

To test our hypothesis that IFN-β secreted by macrophages could hinder CFs reprogramming *in vivo,* we applied a macrophage scavenger, clodronate (CL2MDP), packaged in liposomes to specifically inhibit macrophages infiltration (van Rooijen and Hendrikx, 2010). IF staining with quantitative image analysis showed that MI mice injected with CL2MDP liposomes had significantly fewer macrophages than those in the PBS control group (Fig. 4H) at MI D7, indicating that CL2MDP-containing liposomes effectively depleted macrophages *in vivo*. The qPCR assay also showed that IFN-β expression were decreased by ~3.2-fold in the macrophage-depleted group compared with the PBS group at Day 7 post-MI (Fig. 4I). We subsequently transplanted MGT-expressing MICFs into MI mice hearts and injected CL2MDP through the tail vein on MI D1, D3 or D5 (Fig. 4J and 4K). After 4 weeks post-transplantation, heart samples were harvested. Time course IF analysis showed that the MI D5 was the best beneficial time point for the reprogramming efficiency and survival rate of mice injected with CL2MDP (Figs. 4L, 4M and S6E–G). In these heart sections, 6.8% α-actinin^+^ and ~1.3% cTnI^+^ eGFP-labeled cells could be observed in the injured areas of the macrophage-depleted group, whereas only ~0.3% eGFP-positive cells expressed α-actinin in the PBS control group (Fig. 4L and 4M).

Considering that macrophages are a majority cell type for IFN-β secretion in the injured hearts, we resuspended MICFs infected with MGT in solution containing neutralizing antibody (NAb) targeting IFNAR, which can prevent IFN-β binding to IFNAR receptors, and transplanted the MICFs into the infarction zone of the hearts during MI. Based on the effects of NAb dosage and application timeline observed *in vitro* (Fig. S6H and S6I), we intraperitoneally injected 400 μg NAbs into the infarct zone of postoperative mice on MI D2, D4, and D6 (Fig. 4N). Four weeks after injection, ~7.4% of eGFP-labeled MICFs were reprogrammed into α-actinin^+^ CM-like cells and ~0.8% of eGFP^+^ cells expressed cTnI in heart sections from mice treated with NAb (Fig. 4O and 4P). By contrast, only ~0.2% α-actinin^+^ iCMs were observed in the vehicle-injected control group (Fig. 4O and 4P). Taken together, these results suggested that IFN-β secreted by macrophages could block MICF reprogramming after myocardial infarction in mice.

### STAT1 phosphorylation mediates IFN-induced suppression of cardiac reprogramming

After investigating the upstream factors involved in IFN signaling-mediated suppression of cardiac reprogramming, we next examined the downstream and mechanism of IFN signaling inhibition of cardiac reprogramming. To this end, we first analyzed the numbers of MICFs between sh*Ifnar1* or sh*Ifnar2* infected MICFs and sh*NT* control following cardiac reprogramming to determine if KD *Ifnar1* or *Ifnar2* enhanced reprogramming efficiency was mediated through the regulation of cell proliferation and found no significant difference in MICFs number between *Ifnar1* or *Ifnar2* KD and control group (Fig. S7A). In addition, qPCR assays indicated that the expression levels of proliferation-related genes such as *Aurkb* and *Ki67*, were comparable between groups on Day 5, with modest decrease of *Ki67* expression at Day 10 or *Aurkb* on Day 15 (Fig. S7B). Next, we performed Ki67 and EdU staining experiments to determine if *Ifnar1* or *Ifnar2* KD improved reprogramming efficiency by promoting iCMs proliferation. IF staining showed that almost no Ki67^+^ or EdU^+^ cells co-stained with cTnI, indicating that the increased numbers of iCMs observed in the *Ifnar1* or *Ifnar2* KD groups were not due to cell proliferation (Fig. S7C and S7D). Finally, we asked whether silencing the IFN signaling pathway might simply influence the number of cells successfully transduced by the lentivirus. To test this possibility, we infected cultured *Ifnar2* KD and control MICFs with MGT-HA lentivirus and conducted IF staining one week later. No significant difference was detected in the number of HA^+^ cells between the MGT + sh*NT* and MGT + sh*Ifnar2* groups (Fig. S7E), which indicated that *Ifnar2* silencing does influence the number of cells transduced by the lentivirus.

**Figure 5.**
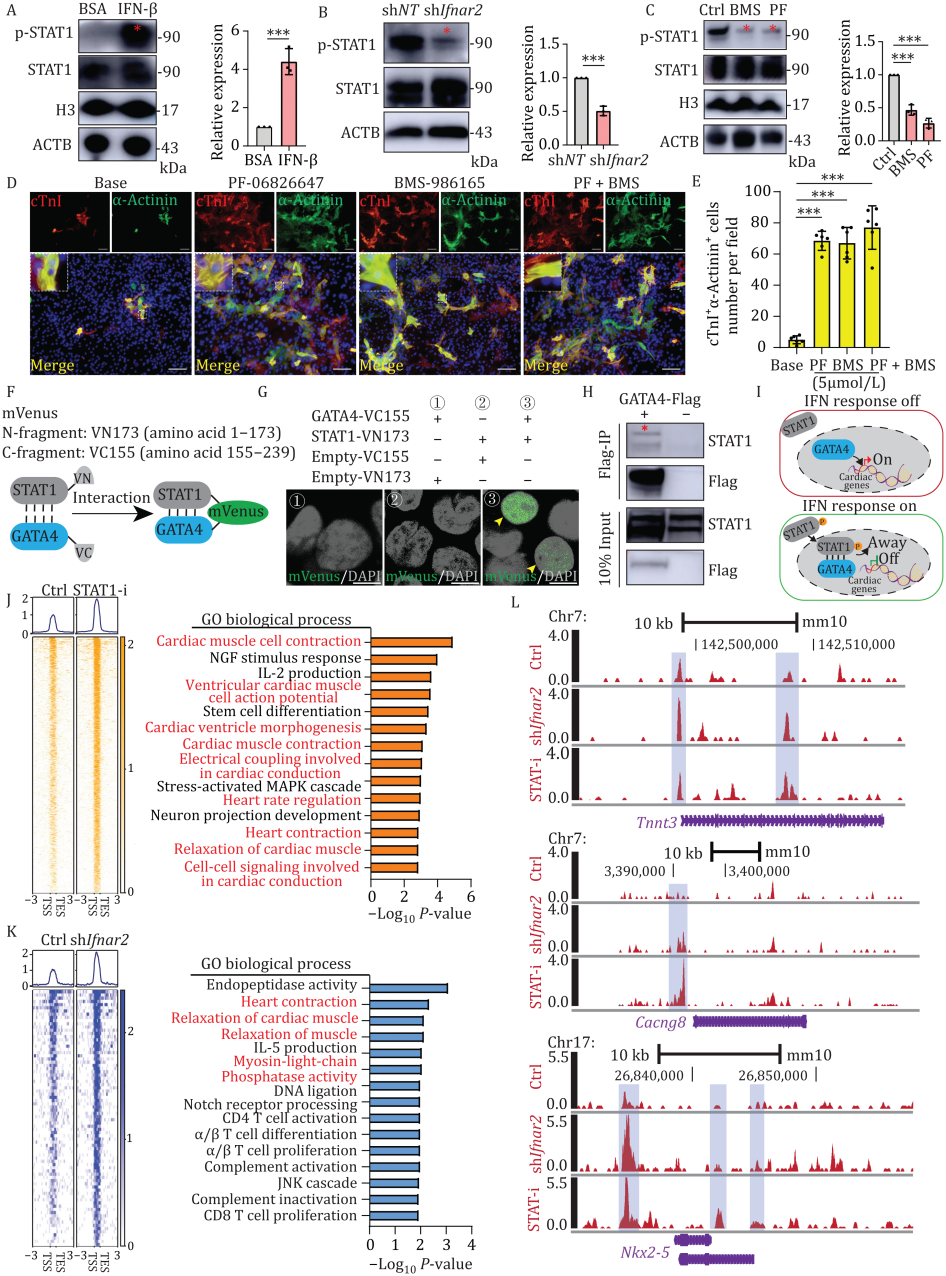
IFN signaling suppresses cardiac reprogramming by STAT1 phosphorylation. (A) Western blot analyses with quantification of p-STAT1 expression in MICFs expressing MGT and treated with 25 IU/mL IFN-β or BSA for 7 days, *n* = 3. (B) Western blot analyses with quantification of p-STAT1 expression in MICFs expressing MGT and sh*Ifnar2* or sh*NT* after 7 days, *n* = 3. (C) Western blot analyses with quantification of p-STAT1 expression in MICFs expressing MGT and treated with DMSO, BMS-986165, or PF-06826647 for 7 days, *n* = 3. (D and E) Representative IF images for α-actinin and cTnI on MGT transduced MICFs treated with DMSO, BMS-986165, or PF-06826647 (D), with quantification of the absolute number in (E). High-magnification views in insects show sarcomeric organization, *n* = 6. (F) Schematic diagram showing the principle of BiFC-mVenus system. (G) BiFC signal results driven by the different combinations of complementation constructs in 293T cells with the treatment of 25 IU/mL IFN-β, *n* = 3. (H) Co-immunoprecipitation assays data for the GATA4-STAT1 interaction. Co-immunoprecipitation assays were performed using MICFs infected with *Gata4*-Flag. (I) Schematic diagram of the hypothesis that p-STAT1 hindered the binding capacity of GATA4 to its targets. In the absence of IFN-β, STAT1 could not be phosphorylated and localized in the cytoplasm, allowing GATA4 bind to cardiomyocyte-associated genes. In response to IFN-β, STAT1 was tyrosine phosphorylated, translocated to the nucleus and complexed with GATA4, trapping GATA4 away from cardiomyocyte-associated genes. (J) CUT&Tag peak heatmap showing GATA4 binding loci in MICFs with treatment of MG(HA)T + sh*NT*/*Ifnar2* + STAT-i or DMSO by using HA antibody (left panel). GO biological process analysis of regions enriched in infected MICFs with the treatment of STAT-i (right panel). (K) CUT&Tag peak heatmap showing GATA binding loci in MICFs with treatment of MG(HA)T + DMSO + sh*Ifnar2* or sh*NT* by using HA antibody (left panel). GO biological process analysis of regions with increased accessibility in infected MICFs with the treatment of sh*Ifnar2* (right panel). (L) IGV tracks showing GATA4 CUT&Tag at *Tnnt3*, *Cacng8* and *Nkx2-5* gene locus between control, STAT-i and sh*Ifnar2* group. All data are presented as the means ± SD. The unpaired *t*-test (A and B) or One-way ANOVA (C and E) was used to determine the significance of differences between two groups. ns, not significant, *P < 0.05, **P < 0.01, ***P < 0.001, Scale bars, 100 μm (G = 8 μm). See also Fig. S7.

IFN-β can reportedly activate STAT1 phosphorylation via binding to the IFNAR receptors (de Weerd and Nguyen, 2012). In the current study, western blot analysis of STAT1 phosphorylation levels after exogenous IFN-β treatment or *Ifnar2* KD showed that phosphorylation of STAT1 was indeed increased in MICFs exposed to exogenous IFN-β (Fig. 5A) but was significantly decreased under knockdown of *Ifnar2* (Fig. 5B). Based on this finding, we proposed that the increase in reprogramming efficiency associated with *Ifnar2* KD could be due to inhibition of STAT1 phosphorylation. To test this hypothesis, we employed two STAT1 phosphorylation inhibitors, BMS-986165 and PF-06826647 (Fig. 5C). After 4 weeks of treatment with inhibitor(s), IF staining assays showed that MGT expression led to higher (~8.2–9.5-fold) induction of cTnI^+^α-actinin^+^ cells with well-formed sarcomere structures in the inhibitor treatment groups compared with that in the DMSO control group (Fig. 5D and 5E). These results suggested that IFN-β suppresses reprogramming in MICFs via the IFN-β-IFNAR-p-STAT1 pathway.

To better understand how phosphorylated STAT1 affects reprogramming, we next examined whether p-STAT1 could interact with the cardiac transcription factor, GATA4, which has been shown to interact with STAT family proteins (Ma et al., 2015; Wang et al., 2005). Specifically, we hypothesized that interaction with phosphorylated STAT1 could inhibit the GATA4 capacity in regulating cardiomyocyte-associated target genes, thus impairing cardiomyocyte fate determination. To visualize cellular localization of STAT1 by fluorescent microscopy we linked the eGFP downstream of the STAT1 gene (STAT1-GFP) and transduced STAT1-GFP into purified MICFs. In response to IFN-β, ~80% of the STAT1-GFP fusion proteins were tyrosine phosphorylated, translocated to the nucleus and colocalized with GATA4 (Fig. S7F and S7G). Bimolecular fluorescence complementation (BiFC) assays to detect potential interactions (Fig. 5F) revealed that overexpression of STAT1‐VN and GATA4‐VC fusion proteins resulted in generating obvious mVenus signal in the nucleus of HEK293T cells with the treatment of IFN-β, but not in the negative control groups (Fig. 5G), suggesting that p-STAT1 could potentially interact with GATA4. Further examination of this possible interaction by co-immunoprecipitation (CO-IP) assays and Western blot confirmed that GATA4 could indeed interact with p-STAT1 in MICFs (Fig. 5H). These cumulative results supported that p-STAT1 could interact with GATA4 in MICFs.

To further validate the hypothesis by which p-STAT1 interacts with GATA4, hindering the GATA4 capacity in binding and transcription in myocardial-related genes (Fig. 5I), we performed the chromatin occupancy profiling of GATA4 in MICFs using CUT&Tag (cleavage under targets and tagmentation) followed by next-generation sequencing in 1 week after treatment with MG(HA)T + DMSO + sh*NT* (referred to as “Control”), MG(HA)T + p-STAT1-inhibitors + sh*NT* (referred to as “STAT-i”), or MG(HA)T + DMSO + sh*Ifnar2* (referred to as “sh*Ifnar2*”). It should be noted that MG(HA)T is a polycistronic construct that allows HA-tagged GATA4 fusion expression. Overall, treatment with p-STAT1 inhibitors or silencing of *Ifnar2* led to an increase in binding peak density of GATA4 compared with control group indicating that the presence of p-STAT1 strongly impacted the genomic occupancy capacity of GATA4 (Fig. S7H). Next, we examined the genomic occupancy pattern of GATA4 in the presence of p-STAT1 inhibition or not following reprogramming. Most binding peaks of GATA4 were enhanced after inhibition of p-STAT1 with STAT-i or sh*Ifnar2* (Fig. 5J and 5K). GO enrichment analysis indicated that most of binding peaks enhanced by p-STAT1 inhibition or *Ifnar2* KD were associated with heart- or muscle-relate (Fig. 5J and 5K). For example, binding peaks associated with the sarcomere genes *Tnnt3* which encode subunits of the troponin complex, calcium channel gene *Cacng8* involved in regulating muscle contraction, and cardiac transcriptional factor *Nkx2-5* were enhanced after p-STAT1 inhibition or *Ifnar2* KD compared with control group (Fig. 5L). These results demonstrated that inhibition of p-STAT1 with STAT-i or sh*Ifnar2* strongly enhanced the binding of GATA4 to its targets.

Next, we sought to understand how p-STAT1 reduces the genomic occupancy of GATA4. Specifically, we hypothesized that p-STAT1 could be complex with GATA4 and trap them away from the myocardial-related genes and localized at STAT1 genomic loci. To this end, we examined the peaks that were enriched in the control group but down-regulated in STAT-i or sh*Ifnar2* group (Fig. S7I). The TRANSFAC and JASPAR PWMs analysis showed that most of these peaks-associated genes were annotated to STAT1 binding genes (Fig. S7J). Gene set enrichment analysis (GSEA) also demonstrated that the STAT1 target genes were enriched in control group compared to those in sh*Ifnar2* or STAT1-i group (Fig. S7K). For example, *Oas2*/*Oas3,* the targets of STAT1, essential genes involved in the interferon response, were enriched in the control group but impoverished after p-STAT1 inhibition or *Ifnar2* KD (Fig. S7L). Motif analyses revealed that ~7.9% of enriched GATA4 target sequences in control group possessed STAT1::STAT2 motif compared to those in sh*Ifnar2* or STAT1-i group (Fig. S7M). Combined with the previous BiFC and Co-IP assay, these results implied a potential of p-STAT1 interacting with GATA4, thereby trapping GATA4 away from its genomic targets and locating to STAT1 genomic loci.

### MICFs recruit macrophages through a positive feedback loop mediated by IFN signaling stimulated CCL2/7/12 expression

**Figure 6.**
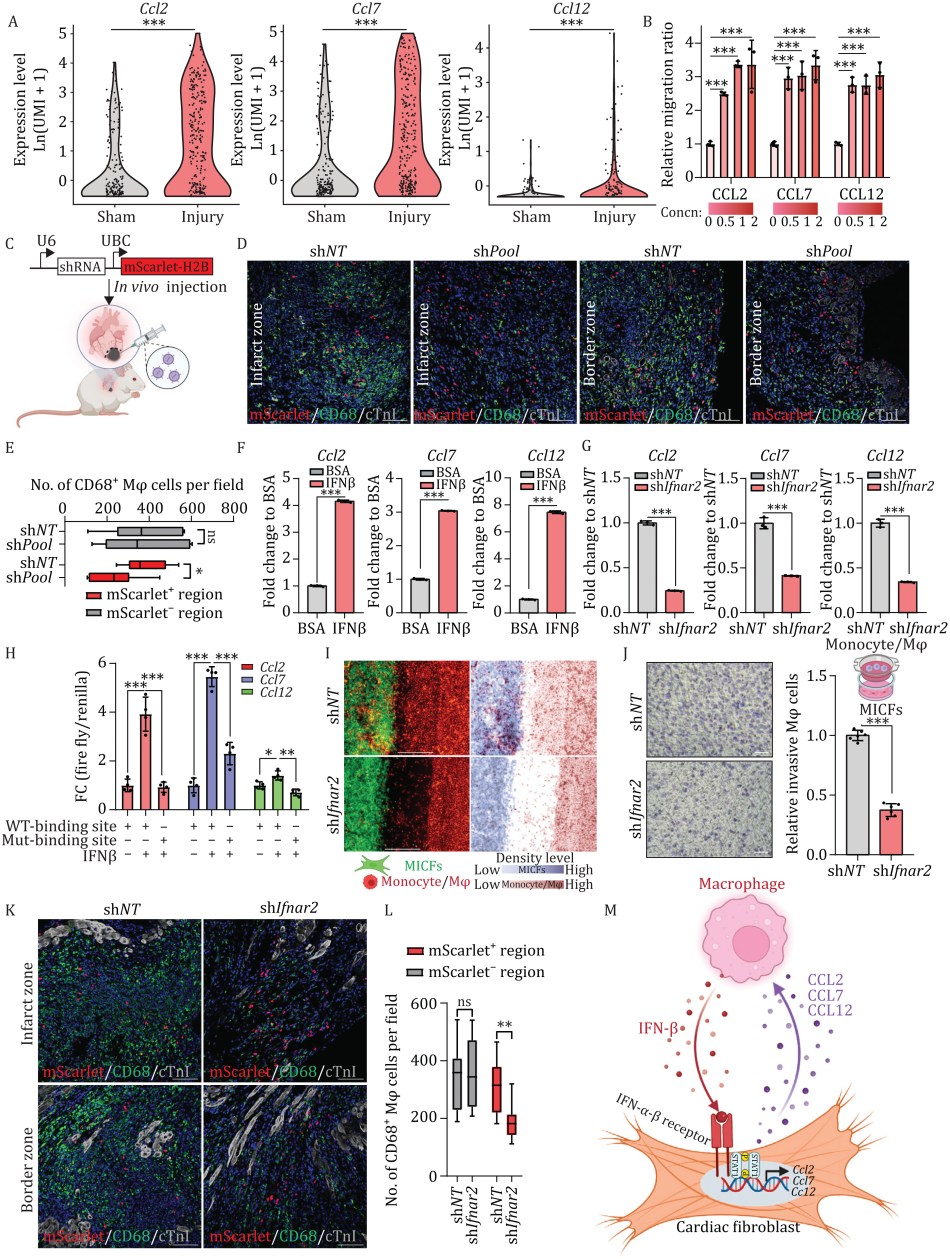
IFN-β responsive MICFs recruit macrophages via a positive feedback loop. (A) Violin diagrams showing the expression of *Ccl2*/*7*/*12* in cardiac fibroblasts under sham or injury condition. (B) Quantification of migration ratio from the monocytes/macrophages transwell migration assay with various chemokines, *n* = 3. (C–E) Experimental workflow (C) and representative IF images for CD68 and mScarlet-H2B on *in vivo* MICFs knockdown experiment (D), with quantification of CD68^+^ macrophages from *in vivo* MICFs knockdown experiment (E), *n* = 3. (F) RT-qPCR analyses for *Ccl2*/*Ccl7*/*Ccl12* genes expression at 3 days after treatment of 25 IU/mL IFN-β or BSA in MICFs, *n* = 3. (G) qPCR-based quantification of *Ccl2*/*Ccl7*/*Ccl12* in *Ifnar2* KD MICFs compared with the sh*NT* group, *n* = 3. (H) Dual luciferase assay in MICFs with indicated promoters, *n* = 4. (I) Representative IF images and density plot images of BMDMs co-cultured with MICFs expressing sh*Ifnar2* or sh*NT*, *n* = 3. (J) Quantification of the BMDMs transwell migration assay using MICFs expressing sh*Ifnar2* or the sh*NT, n = *3. (K) Representative IF images for CD68 and mScarlet-H2B on *in vivo* MICFs knockdown experiment, *n* = 3. (L) Quantification of CD68^+^ macrophages from *in vivo* MICFs knockdown experiment. (M) Schematic diagram of the self-stimulating positive feedback loop between MICFs and macrophages. All data are presented as the means ± SD. The unpaired *t*-test (B, E, F, G, J and L) or one-way ANOVA (B and H) was used to determine the significance of differences between two groups. ns, not significant, *P < 0.05, **P < 0.01, ***P < 0.001, Scale bars, 100 μm (F = 200 μm). See also Fig. S8.

In light of our results showing macrophages secreted IFN-β to activate the IFN-β-IFNAR-p-STAT1 pathway of MICFs and this pathway inhibited cardiac reprogramming. Previous studies have shown that MICFs could produce cytokines participating in inflammatory response after heart injury (Humeres and Frangogiannis, 2019). A recent study (Patel et al., 2018) proposed that chemokines, CCL2, CCL7, and CCL12 can significantly enhance monocytes recruitment in injured heart and the monocytes differentiate into macrophages in the injury site (We call the associated cells as monocytes/macrophages in the following paper.). We next sought to investigate whether MICFs will have a feedback effect on the influx of immune cells after activation of IFN-signaling in the MI microenvironment. Returning to our above analysis of public scRNA-seq data, we found that cardiac fibroblasts highly expressed *Ccl2*, *Ccl7*, and *Ccl12* after heart injury compared to sham (Fig. 6A). This finding led us to speculate that MICFs may participate in the recruitment of monocytes/macrophages via CCL2, CCL7, and CCL12 secretion after MI.

To test this hypothesis, we performed transwell migration assays with monocytes/macrophages isolated from naïve mice and found that exposure to CCL2, CCL7, and CCL12, individually, could induce monocyte/macrophage migration in a concentration-dependent manner (Fig. 6B). To further investigate the role of CCL2/7/12 in the recruitment of monocytes/macrophages *in vivo*, we performed *in vivo* KD experiments via injection of lentivirus expressing sh*NT* or sh*Pool* (sh*Ccl2*/*7*/*12*) tandem with mScarlet-H2B into hearts of MI mice, which could label infected cells with red fluorescence in the nucleus (Figs. 6C and S8A). At 1 week after MI, IF staining assays revealed that significantly more CD68^+^ macrophages were recruited to the injury site in sh*NT* group compared with the sh*pool* group (Fig. 6D and 6E). Next, we sought to investigate whether activation of the IFN signaling pathway would in turn affect CCL2/7/12 secretion in MICFs. As we expected, qPCR analysis showed that stimulation with IFN-β led to ~3.1–7.5-fold higher *Ccl2*, *Ccl7*, and *Ccl12* expression in MICFs compared to untreated controls, while the expression of these chemokines significantly decreased by ~2.5–4.2-fold in *Ifnar2* KD cells compared with that in the sh*NT* control group (Fig. 6F and 6G). In addition, we cloned the promoter sequences of *Ccl2*/*7*/*12* for dual-luciferase promoter activity assays. These dual-luciferase assays showed that IFN signaling could stimulate the activity of each of these promoters to drive *Ccl2*, *Ccl7,* and *Ccl12* transcription (Figs. 6H and S8B). Moreover, lower luciferase signal was detected in cells expressing *Ccl2*/*7*/*12* promoter variants harboring a mutated STAT1 binding sites according JASPAR database (Figs. 6H and S8C). These results suggested that MICFs could recruit monocytes/macrophages by secreting CCL2, CCL7, and CCL12, and the recruited monocytes/macrophages secreted IFN-β, further stimulating phosphorylation of STAT1 which upregulated the expression of these chemokines in MICFs, ultimately forming a self-stimulating positive feedback loop. It was therefore reasonable to propose that inhibition of IFN-β signal transduction in MICFs could suppress monocytes/macrophages recruitment to the injury site.

To explore this hypothesis, we co-cultured BMDMs (isolated from tdtomato-expressing transgenic mice) and MICFs infected with eGFP and sh*Ifnar2-* or sh*NT*-expressing lentiviruses in a single dish separated by a permeable membrane allowing diffusion of signal molecules. After 18 h incubation, we observed that significantly fewer monocytes/macrophages were recruited to the membrane in the sh*Ifnar2* group compared with abundant monocytes/macrophages observed in the sh*NT* group (Fig. 6I), indicating that MICFs could secrete a diffusible signal to recruit monocytes/macrophages which was greatly decreased after *Ifnar2* KD. Further *in vitro* transwell migration assays of BMDMs using MICFs expressing sh*Ifnar2* or the sh*NT* control showed that strikingly higher numbers (~2.7 fold) of monocytes/macrophages were recruited to the chamber containing sh*NT* control MICFs than to the chamber containing MICFs with *Ifnar2* KD, as determined by crystal violet staining (Fig. 6J).

To further investigate the role of MICFs in the recruitment of monocytes/macrophages *in vivo*, we injected lentivirus expressing sh*NT* or sh*Ifnar2* tandem with mScarlet-H2B into hearts of MI mice. At one week after MI, IF staining assays revealed that significantly more CD68^+^ macrophages were recruited to the injury site in sh*NT* group compared with the sh*Ifnar2* group (Fig. 6K and 6L). In addition, qPCR assay of isolated tissue from the infarcted area showed the expression of *Ifnb1* was significantly decreased by ~2.4-fold at Day 7 post-MI and decreased ~1.6-fold at Day 14 post-MI in sh*Ifnar2*-mScarlet-H2B group compared with the control group (Fig. S8D and S8E). These results suggested that *Ifnar2* KD *in vivo* could suppress monocytes/macrophages recruitment to injury site after MI. Overall, these *in vitro* and *in vivo* results described a positive feedback loop of MICFs and monocytes/macrophages recruitment in MI microenvironment, which could be disrupted by *Ifnar2* KD, leading to efficient reprogramming (Fig. 6M).

Taken together, these findings suggested that IFN-β-responsive MICFs recruit monocytes/macrophages after myocardial infarction, and macrophages secreted IFN-β phosphorylate STAT1 in MICFs which further increases the expression of *Ccl2*, *Ccl7,* and *Ccl12*, forming a positive feedback loop. Thus, a self-stimulating IFN signaling circuit mediated by two cell types imposes a major molecular barrier for *in situ* cardiac reprogramming after myocardial infarction, and removal of this barrier by knockdown or chemogenetic inhibition enables the induction of cardiac regeneration through the simple MGT reprogramming factors.

## Discussion

**Figure 7.**
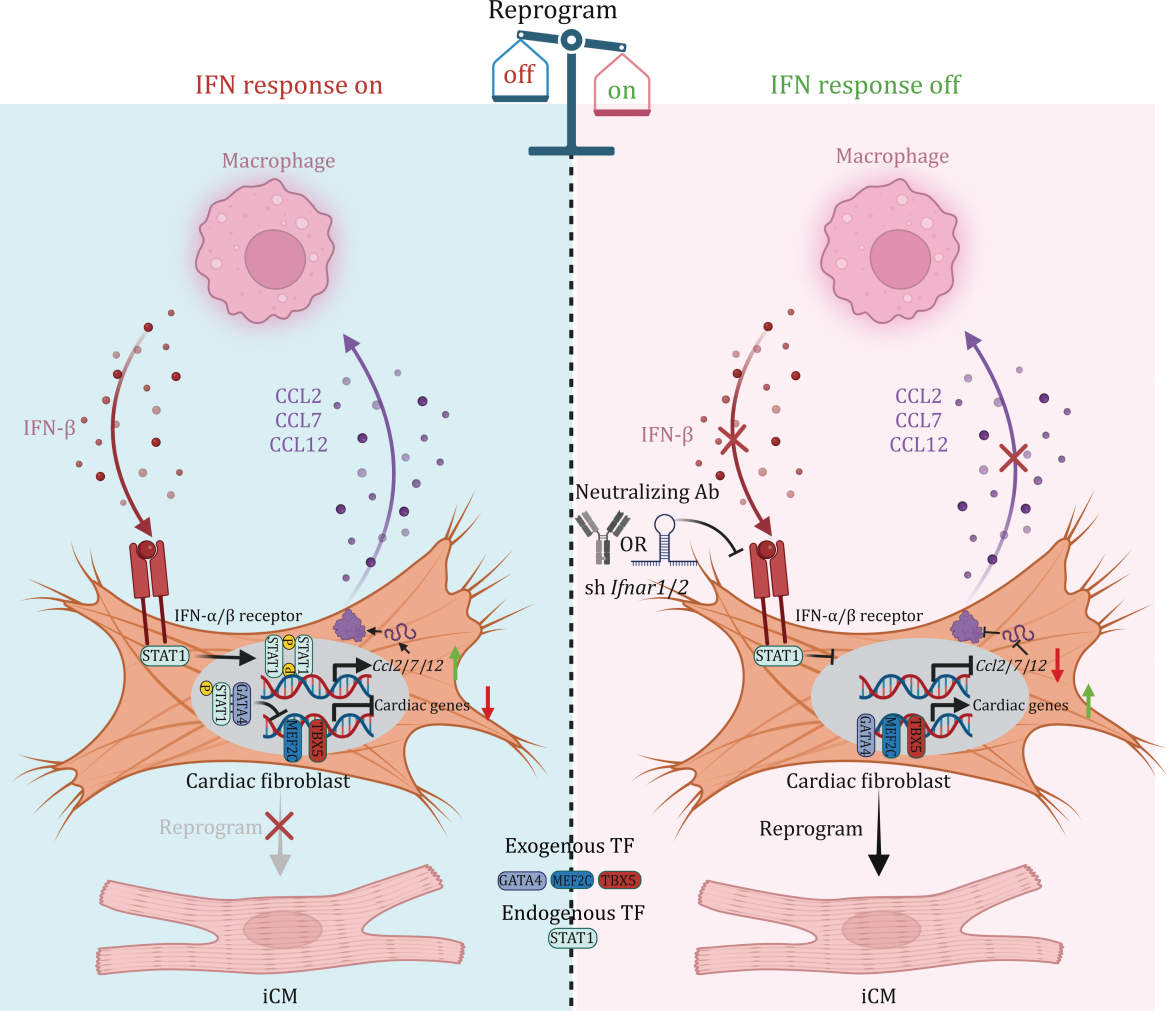
**Macrophages resist cardiac reprogramming *in vivo* via IFN-mediated intercellular self-stimulating circuit.** Macrophages secrete IFN-β, activating the IFN-β-IFNAR-p-STAT1 axis in MICFs, leading to increased secretion of CCL2/7/12 chemokines and subsequent recruitment of more IFN-β-secreting macrophages. These recruited macrophages further enhance STAT1 phosphorylation, establishing a self-reinforcing positive feedback loop between MICFs and macrophages. Knockdown of IFNAR disrupts this loop, facilitating efficient reprogramming.

In this study, we depict the role of macrophages in suppressing cardiac reprogramming via IFN-β-IFNAR-p-STAT1-CCL2/7/12 axis in a feedforward system between MICFs and macrophages and demonstrate that macrophages function as a key niche cell inhibiting cardiac reprogramming. In the MI microenvironment, macrophages secrete IFN-β, which activates the IFN-β-IFNAR-p-STAT1 axis of MICFs. The upregulation of IFN-IFNAR-p-STAT1 signaling could stimulate MICFs secretion of CCL2/7/12 chemokines, subsequently recruiting IFN-β-secreting macrophages. These recruited macrophages further activate STAT1 phosphorylation, translocating to the nucleus, improving CCL2/7/12 secretion and immune cell recruitment, ultimately forming a self-reinforcing positive feedback loop between MICFs and macrophages *in vivo*. Knockdown of *Ifnar1* or *Ifnar2* in MICFs prevents p-STAT1 accumulation in the nucleus, thereby enhancing the genomic occupancy capacity of GATA4 to cardiomyocyte-associated genes (Fig. 7).

*In situ* reprogramming after injury is particularly challenging in cell types that cannot regenerate after differentiation is complete, such as neurons and cardiomyocytes. Recent studies in mTmG labeled mice (Isomi et al., 2021) to exclude the possibility of Sendai virus-mediated CFs-CMs fusion or transgenic mice expressing HMGT (Tani et al., 2023) both showed extensive cardiac reprogramming *in vivo*. However, even relatively uniform high expression of HMGT led to only ~2.0% *in situ* reprogramming efficiency in transgenic mice, which was markedly lower than *in vitro* efficiency, and implied that *in vivo* factors might be antagonizing the effects of the four transcription factors.

In 2012, Inagawa et al. reported that the infected cells would be eliminated by immune cells after delivering retrovirus into the heart in mice (Inagawa et al., 2012). Although *in vivo* studies have subsequently relied on immunodeficient or genetically modified mice for a decade since that discovery, *in vivo* reprogramming efficiency has remained low. To overcome immune rejection and identify molecular barriers to reprogramming in wild-type mice, we developed the TMM (Tacrolimus, mycophenolate mofetil, and methylprednisolone) immunosuppressant cocktail to attenuate immunologic response, facilitating direct virus injection and allogeneic cell transplantation *in vivo*. With the administration of TMM, we achieved long-term retention of transplanted cells (retention rate: ~7.6%, 4 weeks), consistent with previous reports (Li et al., 2021a). Moreover, the use of transplantation system in methodology resulted in greatly reducing inter-batch variations caused by infection and enabled the refined comparison of reprogramming at the injury microenvironment *in situ* and *ex vivo*.

Mechanistically, the complex formed by Tacrolimus and FKBP inhibits calcineurin phosphatase and the proliferation of T cells (Thomson et al., 1995). Mycophenolate mofetil can deplete guanosine in lymphocytes to inhibit their proliferation and can also decrease the influx of lymphocytes and monocytes in inflammatory areas via inhibiting the glycosylation and expression of adhesion molecules (Allison, 2005). Methylprednisolone binds to glucocorticoid receptors to affect the expression of inflammatory factors in immune cells (Auphan et al., 1995). In general, the use of TMM cocktail will decrease the recruitment and activation of immune cells in the injury area of mice. However, probably due to excessive inflammation, the number of immune cells and expression of IFN-related genes decreased slightly (macrophage counts were comparable and T-cell counts with a significant decrease) in TMM treated group compared with vehicle treated group in the injured area of mice but were still significantly higher than those in the sham group (Fig. S8F–H), suggesting that the administration of TMM negligibly affect the impact of IFN signaling pathway on MICFs in the MI microenvironment. Combined with the previous *in vitro* macrophage supernatant addition assay (Figs. 4F and S6D), macrophage supernatant secretions can also inhibit the conversion of MICFs to cardiomyocytes in the absence of TMM immunosuppressant administration, suggesting that increased reprogramming efficiency is due to blocking macrophage-mediated IFN signaling rather than the use of TMM immunosuppressant.

Here, we found that the inflammatory microenvironment plays a vital role in cardiac reprogramming *in vivo* and that reprogramming involves a complex intercellular communication network to restore functionality in heart tissue. Previous publications also showed that inflammatory microenvironment influences cell fate determination (Mosteiro et al., 2018; Palacios et al., 2010). In cardiac regeneration, studies in zebrafish have shown that macrophages secrete erythropoietin to promote cardiac regeneration and that IFN-γ promotes heart regeneration via activation of estrogen signaling (Xu et al., 2020). In 2017, Zhou et al. (2017) identified that ZNF281 enhanced cardiac reprogramming by suppressing inflammatory gene expression. Over the past decade, several papers have reported the role of inflammatory pathways in cardiac reprogramming with chemogenetic inhibition *in vitro* (Guo et al., 2019; Hashimoto et al., 2019; Hodgkinson et al., 2018; Jayawardena et al., 2012; Muraoka et al., 2019; Zhou et al., 2015, 2017). However, these studies indicated the inhibitory effects of inflammation only in MEFs or neonatal cardiac fibroblasts in culture conditions, without microenvironment stimulation or immune cell communication, and the mechanism remains unclear. In comparison, our results suggest a concept that macrophage in MI microenvironment may directly affect lineage reprogramming *in situ* in mammals and demonstrate that inflammation pathway suppress cardiac reprogramming via inhibiting the binding of GATA4 to myocardial-related genes by p-STAT1.

Our current study provides, to the best of our knowledge, the first report of a feedback loop regulating immune cell recruitment and activity in the post-MI environment involving fibroblasts and macrophages, mediated by the IFN signaling pathway. MI involves physiological changes in many cell types, and it is generally accepted that damage-associated molecular patterns (DAMPs) act as triggers for macrophage recruitment, causing an inflammatory response around myocardial infarction area (Frangogiannis, 2014; King et al., 2017). Previous studies have shown that CCL2/7/12 can stimulate monocyte/macrophage infiltration at the site of heart injury (Patel et al., 2018). In this study, we found that cardiac fibroblasts can further recruit monocytes/macrophages by secreting CCL2, CCL7, and CCL12 cytokines. Other chemokines such as CCL8/11/13/26, MSMP, GMCSF et al. have also been reported to be involved in macrophage recruitment (Bai et al., 2023), but in our study, these chemokines were not significantly up-regulated after IFN-β stimulation. In addition, previous studies have shown that recruited macrophages can recruit CXCR3^+^ T cells by secreting CXCL10 in injured heart (Ngwenyama et al., 2019). And T cells, particularly Th1 CD4^+^ and CD8^+^ T cells can secrete IFN-γ to activate STAT1 phosphorylation through the IFNGR receptor (de Weerd and Nguyen, 2012; Grabie et al., 2007). In light of these reports, we speculate that there may be a bigger loop among MICFs, macrophages, and T cells. Disruption of this self-stimulating IFN-mediated inflammatory circuit or macrophages depletion would not only remove molecular barriers to reprogramming to enable *in situ* cardiac reprogramming but also attenuate the excessive recruitment of immune cells and alleviate severe inflammation at the site of MI.

Growing evidence in the field suggests that the improvement in heart function is not solely attributable to the generation of new iCMs in the damaged myocardium. In addition to the reprogramming of MICFs into iCMs, attenuating the excessive immune response by blocking IFN pathway and altering fibroblast behavior may also contribute to ameliorated heart function. It has been reported that the recruited macrophages induced an IFN response in cardiomyocytes promoting adverse ventricular remodeling, a common antecedent of heart failure (Yerra et al., 2023). A recent study employed extracellular matrix-nanostructured to localized delivery of anti-inflammatory agents to promote cardiac repair after myocardial infarction (Wang et al., 2023). Further studies used IFN-γ blockade or small molecular suppressing macrophage hyperactivation to ameliorate heart function after injury (Decano et al., 2023). During the review of this article, a study of *in vivo* cardiac reprogramming by GHMT-genetically modified mice was published indicating that cardiac reprogramming reduces inflammatory macrophages and improves cardiac function in chronic myocardial infarction, implying the negative role of macrophages in cardiac repair (Abe et al., 2023). Tani et al. utilized single-cell RNA-seq indicating that the profibrotic cardiac fibroblasts were converted to quiescent fibroblasts that had a transcriptional profile more similar to the uninjured state with the treatment of reprogramming factors cocktail (Tani et al., 2023). Moreover, it has been demonstrated that quiescent cardiac fibroblasts protect cardiomyocytes from ferroptosis through paracrine factors and direct cell-cell interaction (Mohr et al., 2023). Hence, the observed reduction in scar size and improved heart function in the MGT + sh*Ifnar2* group may be attributable to the synergy effects of iCMs regeneration, immunomodulatory, protection of cardiomyocytes (from fibroblasts or other non-myocytes via paracrine effects) and attenuation of adverse cardiac remodeling.

In the current work, we illustrate the prominent role of the IFN-β-IFNAR-p-STAT1 signaling pathway in regulating cardiac cell fate determination. Recently, other studies reported that ZNF281 (Zhou et al., 2017) and PHF7 (Garry et al., 2021) could physically interact with GATA4 to upregulate still more myocardial-related genes and promote the conversion of fibroblasts. In comparison, our findings show that phosphorylated STAT1 can physically interact with GATA4, hindering its binding and transcription in myocardial-related genes. To date, previous investigations in cardiac reprogramming have primarily focused on identifying and overcoming obstacles related to fibrosis signaling, such as the TGF-β (Knott et al., 2014) (which plays a key role in activating myofibroblast cell fate), or factors that affect epigenetic modification, such as *Bmi1* (Zhou et al., 2016). Based on our findings in this study, we propose that molecular barriers may negatively affect cell fate conversion by physically binding to the reprogramming transcription factors and hindering transcription of their downstream genes.

In summary, by comparing RNA-sequencing data from *in vitro* and *in vivo* samples during cardiac reprogramming, we revealed that disrupting the IFN-β signaling pathway or depleting macrophages can down-regulate STAT1 activation, and facilitate the reprogramming-based cardiac regeneration. Moreover, we also identified a large suite of differentially expressed genes that might also suppress cell fated conversion, indicating the need for further exploration. Combining factors identified in this study and others may ultimately lead to a safe and reliable reprogramming strategy for therapeutic clinical regenerative medicine applications.

## Methods

### Mice and surgery

Eight-week-old male wild-type (WT) C57BL/6 mice were purchased from Peking University Animal Center. *Col1a2*-*CreER*^+/−^, *Tcf21*-*CreER*^+/−^ mice obtained from National Institute of Biological Sciences (NIBS), were used to trace cardiac fibroblasts by crossing with *Rosa26*-*lsl-tdTomato*^+/+^ mice. *R26*-*mTmG* mice were a gift from Dr Bo Shen (NIBS). Transgenic mice overexpressing tdTomato protein purchased from Beijing Vitalstar Biotechnology. The animal protocol for surgery followed the institutional guidelines and was approved by PKU Institutional Animal Care and Use Committee. Myocardial infarction (MI) surgery was induced by permanent ligation of the left anterior descending artery (LAD) as described by (Virag and Lust, 2011). Briefly, 8 weeks old male C57BL/6 mice were anaesthetized with 2.5% isoflurane/97.5% oxygen and placed in a supine position. Animals were intubated with a 24 G stump needle and ventilated with 1.5% isoflurane/98.5% oxygen using a VentElite mouse ventilator (Harvard Apparatus). MI was induced by permanent ligation of the LAD with a 6-0 nylon suture.

### Isolation and culture of MICFs

For isolation of MICFs, hearts were excised from C57BL/6 mice with MI surgery on Day 6 to ensure the isolated cardiac fibroblasts stayed in the cell identity of myofibroblasts (Fu et al., 2018). The infarcted area of heart was dissected from surrounding normal myocardium and chopped with razor blades into small pieces (≤1 mm^3^). The chopped samples were digested in 2 mL collagenase I/II/IV (Gibco, 2 mg/mL) with Dispase II (Sigma, 2 mg/mL) and incubated at 37°C in 6 well plates for 2 h. After digestion, the cells were filtered through a 70 µm and 40 µm cell sieve. Then centrifuged and added red blood cells lysis buffer (Solarbio) to remove red blood cells. After being centrifuged and resuspended, MICFs were plated in 24-well plates with culture medium (DMEM supplemented with 10% FBS and 2% penicillin/streptomycin).

For MICFs purification, the MICFs were incubated with CD45 beads for 20 min at 4°C. After being the centrifuged and resuspended according to MACS instruction, MICFs were plated in 24-well plates with culture medium.

### Plasmid construction and virus packing

For construction of doxycycline inducible polycistronic reprogramming vector, the polycistronic cassette MGT cloned from pMX-MGT (Addgene, #111810) vectors were inserted into the Fu-tet-O vector (Addgene, #19778) behind the tet operator sites and minimal CMV promoter (Tet-on system). Similarly, pFu-tet-o lentiviral vector contain *Myocd*, *Sall4* or eGFP were generated as described (Zhao et al., 2021). All shRNAs sequence listed in Table S1 were cloned into the pLKO.1 HIV-based lentiviral vector (pLKO.1-TRC control, Addgene, #10879) using the AgeⅠ and EcoRⅠ restriction enzyme sites. pLKO.1-shRNA-UBC-H2B-mScarlet vector were generated via subcloning H2B-mScarlet sequence and UBC promoter sequence into pLko.1-shRNA vector digested with KpnⅠ and SacⅠⅠ restriction enzyme using Gibson Assembly (Transgene, CU101-03). The pFu-U6-shRNA-UBC-rtTA used *in vivo* cardiac reprogramming were generated through inserted the amplified sequence U6-shRNA into the pFUdeltaGW-rtTA (Addgene, #19780) vector digested with AleⅠ and PacⅠ restriction enzyme according to the Gibson ligation manufacturer’s instructions. The lentivirus packaging and envelop vectors, pMDLgpRRE (Addgene, #12251), pRSV-Rev (Addgene, #12253) and pVSV-G (Addgene, #138479) obtained from Addgene. Lentivirus packaging was performed in HEK293T cells maintained in DMEM growth media containing 10% FBS. One day before transfection, HEK 293T cells were plated in 10 cm dishes at a density of 8 × 10^6^. The next day, 15 µg targets vector (shRNA, pFu-tet-O etc.), 5 µg pMDLgpRRE, 5 µg pRSV-Rev and 5 µg pVSV-G were gently mixed with 50 μL of 2.5 mol/L CaCl_2_ and 450 μL ddH_2_O then dropped DNA/CaCl_2_ mix to 500 µL 2× HBS solution immediately, adding HBS/DNA solution onto cells for transfection. Supernatant was collected 48 h post-transfection and filtered through 45 µm pore size filters.

### Cardiac reprogramming *in vitro*

MICFs were infected by freshly lentiviruses (5 × 10^6^ TU/mL) with 8 µg/mL polybrene at a density ~90% confluence on 24-well plates. Twenty-four hours later, MICFs culture medium were changed to iCMs media (4:1 DMEM:M199 with 10% FBS, 10% KSR, 1% P/S, 1% NEAA, 1% Glutamax, 2 μmol/L SB431542 and baricitinib, 2 μg/mL doxycycline) (Tao et al., 2023) which was changed every 2 days.

### Cell transplantation

MICFs at a density ~90% confluence were infected with lentivirus and harvested 2 days after infection. A left thoracotomy was carried out in C57BL/6 mice, and 10^6^ cultured cells were injected into the left ventricle immediately after left anterior descending ligation. To reduce the immune response, mice were treated with TMM (2 mg/kg/day Tacrolimus, 20 mg/kg/day methylprednisolone and 20 mg/kg/day mycophenolate mofetil) by intraperitoneal injection for 2 days per day before surgery. The TMM immunosuppressants were injected every day until analyses. 2 mg/mL doxycycline with 5% sucrose was added to drinking water to drive the gene expression before surgery until harvest.

### *In vivo* gene delivery and CreER induction

High-titer virus-containing (5 × 10^6^ TU/mL) supernatants were collected, filtered through 0.45 µm pore membranes, and add polybrene with a final concentration of 8 µg/mL. Supernatants were concentrated 1000-fold with centrifugation (50,000 ×*g* for 2 h at 4°C), and then resuspended in phosphate-buffered saline. For animal function experiments, 25 µL of pooled concentrated high-titer viral (pFu-tet-MGT-HA/pFu-U6-shRNA-Ubc-rtTA) supernatant were immediately injected into the boundary between the infarct and border zone after the coronary artery ligation. In the cardiac reprogramming experiments, 25 µL of pooled virus solution (pFu-tet-MGT-HA/pFu-U6-shRNA-Ubc-rtTA) was administered during MI. Consistent with cell transplantation, mice were treated with TMM 2 days before virus delivery and continued until harvest. Drinking water was added with 2 mg/mL doxycycline and 5% sucrose to drive the gene expression before surgery. For lineage tracing experiments, tamoxifen (Sigma, T5648, 45 mg/kg/day) was administered to the mice by intraperitoneal injection for five consecutive days (Tani et al., 2023). Mice were subjected to surgery on Day 7 post-administration of tamoxifen (Sigma, T5648). Tamoxifen (Sigma, T5648) was dissolved in corn oil (90%) and ethanol (10%) at a concentration of 50 mg/mL.

### Echocardiography

Cardiac function after myocardial infarction surgery was obtained by transthoracic echocardiography (Visual Sonics, Vevo 2100). To ensure all mice experienced a similar degree of surgical injury, mice with an ejection fraction greater than 50% were eliminated and detected on day 2 after MI. Mice underwent echocardiography at 4 weeks after MI. Mice were depilated the day before echocardiography. The mice were continuously anesthetized with isoflurane gas during the ultrasound examination, and the heart rate was not lower than 400 bpm during the examination. After obtaining the B mode data of the long axis of the mouse heart, we used the Vevo strain module of the Vevo workstation for data processing to calculate the cardiac function parameters such as EF and GLS of the mouse.

### Immunofluorescence stain and flow cytometry

For immunofluorescence, cells were fixed with 4% paraformaldehyde for 15 min, and permeabilized with PBS buffer containing 0.1% Triton-X for 10 min and blocked with 3% normal donkey serum for 1 h at room temperature. Cells were stained with primary antibody against cTnI (Abcam, ab56357, 1:100), α-actinin (Sigma, A7311, 1:500), Vimentin (Abcam, #ab92547, 1:500), CD31 (BD, #557355, 1:100), CD68 (Abcam, #ab125212, 1:500), CD3 (Invitrogen, #14-0032-81, 1:100), GATA4 (Santa, #sc-25310, 1:100), p-STAT1 (Cell signaling Technology, #9167s, 1:100), HA (Cell signaling Technology, #3724s, 1:500) or Ki-67 (Cell signaling Technology, #9449, 1:400) at 4°C overnight. After washes with PBS, cells were incubated with DAPI and secondary antibodies anti-goat Alexa fluor 555 (Invitrogen, A32816, 1:1000), anti-rabbit Alexa fluor 488 (Invitrogen, A32790, 1:1000) for 1 h at room temperature. Images were captured by inverted fluorescence microscopes (AXIO Vert.A1) and Cell Discovery 7 from Zeiss. For the quantification, four independent experiments were used for scanning whole wells and analyzed in Image J software, or 5–9 fields were randomly selected in a blinded manner and the indicated cells were counted manually in each experiment.

For detecting α-actinin and cTnI expression by FACS, the cells were fixed with 4% PFA for 15 min, permeabilized with saponin (Sigma Aldrich, 47036-250G-F), stained with cTnI (Abcam, ab56357, 1:100), α-actinin (Sigma, A7311, 1:500), followed by incubation with the secondary antibody conjugated with Alexa Fluor 488 and 555. The cells were then analyzed using CytoFLEX (Beckman Coulter).

Mason staining was performed on paraffin-embedded sections. To determine the scar size, we used ImageJ software to measure the scar area (blue) and healthy area (red) on transverse sections spanning five levels. The measurements and calculations were conducted in a blinded manner.

For FACS isolation of eGFP^+^cells, hearts were excised from C57BL/6 mice transplanted with MICFs expressing MGTMS and eGFP and chopped with razor blades into small pieces (≤1 mm^3^). Subsequently, the chopped samples were digested and filtered through a 70 µm and 40 µm cell sieve to obtain single-cell suspensions. Then eGFP^+^ cell isolation was performed on a BD FACSAria3. For FACS quantification analysis of BMDM, the cultured cells were collected and incubated on ice for 15–30 min with anti-mouse CD11b (Biolegend, #101211, 1:300) and anti-mouse F4/80 (Biolegend, #123109, 1:300) for macrophage detection analyzed on a CytoFLEX (Beckman Coulter).

### Tissue slice immunofluorescence

Hearts were fixed in 0.4% paraformaldehyde for 4 h and dehydrated overnight in 30% sucrose. Then embedded in OCT (Macgene) for freezing in liquid nitrogen. Hearts were cut vertically into 10 µm sections. Sections were stained with primary antibodies against α-actinin (Sigma, #A7311, 1:500), cTnI (Abcam, #ab56357, 1:100), GFP (Abcam, #ab13970, 1:500), HA-Tag (CST, #3724s, 1:500), Vimentin (Abcam, #ab92547, 1:500), or CD68 (Abcam, #ab125212, 1:500) and then with secondary antibodies conjugated with Alexa 488, 555, 647, and DAPI. All sections were captured by an A1R confocal microscope (Nikon).

### RT-qPCR assay

Total RNA of MICFs was isolated with TRIzol (Thermo Fisher Scientific) followed by extraction using the Direct-Zol RNA Miniprep Kit (Zymo Research, R2062). For cDNA synthesis, 1 μg of RNA was reverse-transcribed using Oligo-dT (Vazyme, R333-01). qPCR was conducted using Real-Time PCR system (q225, Kubo Tech) with SYBRR Green PCR Master Mix (Vazyme, Q321-03), and expression levels were normalized to the expression of *Gapdh*. The primers listed are shown in Table S2.

### *In vitro* transwell migration and wound healing assay

Transwell migration of monocytes/macrophages was performed with bone marrow-derived macrophages (BMDMs) and MICFs. In brief, bone marrow was isolated from WT mice. After lysing with red blood cell lysis buffer and centrifuged, cells were cultured in fresh DMEM medium containing 10% FBS, 1% P/S, and 10 ng/mL MCSF for 5 more days, after which most of the cells in the culture were BMDM. To test the ability of MICFs to recruit monocytes/macrophage, BMDMs (1.5 × 10^4^) suspended in 100 μL culture medium were placed in the upper chamber and MICFs expressing sh*Ifnar2*/sh*NT* suspended in 600 μL culture medium were placed in the lower chamber. At 18 h after incubation, the migrated BMDMs were stained with 0.1% crystal violet from the lower chambers and statistical quantification. To test the ability of secreted chemokines to promote monocyte migration, CCL2, CCL7, and CCL12 (MCE) at concentrations of 0, 0.5, 1, and 2 μg/mL were added to the culture medium to induce monocyte migration, the migrated monocytes were quantitatively analyzed in 24 h.

In wound healing assay, MICFs infected with EGFP and sh*Ifnar2*/*NT* and BMDMs isolated from *Rosa26*-tdTomato^+/+^ mice were seeded in culture-inserts (ibidi, #81167) at a density of 50,000 cells per well in the presence or absence of Dox (2 μg/mL). After 24 h, the culture-insert was gently removed, and cells were imaged directly after removing the culture-insert for 8–12 h.

### Co-culture experiments

For BMDMs and MICFs co-culture experiments, BMDMs isolated from WT mice and transfected with dsDNA with lipofectamine for stimulation (King et al., 2017). Five days later, the supernatant was collected and formulated into reprogramming medium added to MICFs expressing MGT and sh*Ifnar2*/*NT.*

### ELISA assay

Twenty-hours later the treatment of dsDNA, the supernatant of WT/*Ifnb1*-KO BMDM were collected and assessed using high-sensitivity ELISA kit according to the instructions (Solarbi, SEKM-0032).

### Co-IP and Western blot analyses

For Co-IP experiments, MICFs expressing GATA4-FLAG were lysed with lysis buffer (RIPA, Thermo Fisher Scientific, #89900; 0.2 mmol/L PSMF, Beyotime, #ST506; 0.5 mmol/L DTT Thermo Fisher Scientific, #D1532). Subsequently, agarose-conjugated FLAG antibody (Sigma, #A2220) was washed twice with lysis buffer at 1000 rpm for 3 min and rotated incantation with lysates at 4°C for 3 h. After washing twice with lysis buffer, the lysates were added 4× loading buffer and boiled in at metal bath for 5 min and run on SDS-PAGE gels to separate proteins prior to the immunoblot analyses. After transfer to 0.45 μm PVDF membranes (Millipore, IPVH07850), immunodetection was performed using antibodies specific to STAT1 (HUABIO, #R1408-2, 1:500), FLAG^®^ M2-Peroxidase (HRP) (Sigma-Aldrich, #A8592, 1:3,000) followed by incubation with the HRP-conjugated anti-rabbit IgG secondary antibody. The antibody-bound proteins were visualized by chemiluminescence detection.

For Western blot assay, the whole-cell lysates were prepared using RIPA buffer (1 mmol/L PMST, 1 mmol/L protease inhibitor cocktail, 1 mmol/L DTT, and 1 mg/mL phosphatase inhibitors) and loaded for SDS-PAGE and transferred to PVDF membranes using a Bio-Rad transfer apparatus. Subsequently, the membrane was blocked with 5% nonfat milk in TBS containing 0.1% Tween-20 (TBST) at room temperature for 2 h, followed by incubation with primary antibody overnight at 4°C. The P(Tyr-701)-STAT1, STAT1, H3, and β-actin proteins were detected using the antibodies against P-STAT1 (Cell Signaling Technology, #9167, 1:500), STAT1 (HUABIO, #R1408-2, 1:500), β-actin (ABclonal, #AC004, 1:5,000), and H3 (Abcam, #ab1791, 1:400) followed by incubation with the HRP-conjugated anti-rabbit or mouse IgG secondary antibody. The antibody-bound proteins were visualized by chemiluminescence detection.

### Dual-luciferase assay

The promoter sequence (upstream of TSS ~2 kb) of *Ccl2*, *Ccl7*, *Ccl12* were PCR amplified and cloned into the pGL3-lentiviral vector, respectively. The target sites of STAT1 were predicted through JASPAR database and the mutant promoters were obtained by mutating the STAT1 target sites. For the luciferase reporter assay, MICFs were infected with related virus for 24 h and maintained throughout the culture for an additional 2 days. After 3 days of puromycin/ Zeocin screening, a Dual-Luciferase Reporter Assay Kit (YEASEN, #11402ES60) was used to measure Renilla and Firefly activity according to the manufacturer’s instructions.

### EdU labeling assay

For assessing cell proliferation, 10 μmol/L EdU (Beyotime, Cat# C0071) was added to the iCM medium after 2 weeks of virus infection and maintained throughout the culture for an additional 2 weeks. Cells were fixed in 4% PFA for 10 min followed by permeabilization in PBS/0.1% Triton X-100 for 15 min at room temperature and blocked in blocking buffer for 1 h. Then cells were incubated with primary antibody against cTnI overnight followed by incubation with the Alex Flour 555 secondary antibody. Next, cells were added to click reaction solution to determine Edu using BeyoClick™ EdU Cell Proliferation Kit (Beyotime, Cat# C0071) according to the manufacturer’s instructions.

### Ca^2+^ imaging

Ca^2+^ imaging of beating cells was performed according to the manufacturer’s protocol (Thermo Fisher Scientific, #F10489). Briefly, iCM expressing MGT and sh*Ifnar1*/*2* were added 2 mL of Fluo-4, AM loading solution, and incubated at 37°C for 30 min, followed by 15–30 min at room temperature. Subsequently, Fluo-4 was removed and iCM was washed once with PBS for live-cell imaging. Ca^2+^ oscillations video captured with a fluorescence microscope.

### Analysis of online scRNA data

Mouse heart single-cell expression data analysis was available from Gene Expression Ominbus (GSE120064).

### Bulk RNA sequencing, analysis, and visualization

RNA from FACS-purified eGFP positive cells and couture cells was submitted to Novogene for quantification, RNA-seq library preparation, sequencing, and mapping. Differential gene expression analysis was performed using the DESeq2 package. Genes with significantly upregulated expression (*P *< 0.01, fold change > 2) were chosen for further analysis. Heat maps showing differentially expressed genes were generated using the “pheatmap” and “ggrepel” packages in *R*. Gene-set enrichment analysis (GSEA) was completed with the “ClusterProfiler” Bioconductor *R* package using the default settings and analysis results were visualized by the “enrichplot” Bioconductor *R* package.

### CUT&Tag assay

CUT&Tag was performed essentially as described by Li et al., (2021b). In brief, 0.1 million cells were collected for each sample. The following primary antibodies were used rabbit anti-HA antibody HA-Tag (CST, #3724s, 1:500). Goat anti-Rabbit antibody (1:100; Vazyme) was used as the secondary antibody. pAG-Tn5 was purchased from Vazyme and used for each CUT&Tag reaction building a library according to the Hyperactive Universal CUT&Tag Assay Kit for Illumina Pro protocol (Vazyme, #TD904, Nanjing, China). CUT&Tag library was submitted to ANOROAD for quantification, sequencing, and mapping.

### Quantification and statistical analysis

All experimental data were presented as the mean ± SD. “*n*” represented the number of animals or samples and was indicated in the figure legends. For statistical evaluation, unpaired Student’s *t*-test or One-way ANOVA followed by the Dunnett multiple comparisons test (to a single control group) or the Tukey multiple comparisons test (among groups) were used to determine the difference between groups. For multiple group comparisons with >2 variables the Two-way ANOVA followed by Tukey’s multiple comparisons test was performed using Graphpad Prism software, as indicated in figure legends. Differences with *P* values < 0.05 were regarded as significant.

## Supplementary Material

pwae013_suppl_Supplementary_Material

pwae013_suppl_Supplementary_Movies_S1

pwae013_suppl_Supplementary_Movies_S2

pwae013_suppl_Supplementary_Movies_S3

pwae013_suppl_Supplementary_Movies_S4

## Data Availability

All RNA-seq and Cut-tag data were deposited in Gene Expression Omnibus database under accession number GSE261309. Other data supporting the results can be found in this paper and its supplementary materials.

## Code availability

All data analyses were followed by standard programs and codes are available on reasonable request. All diagrams are drawn by Biorender.
