# Correction to: Macrophages suppress cardiac reprogramming of fibroblasts *in vivo* via IFN-mediated intercellular self-stimulating circuit

**DOI:** 10.1093/procel/pwae038

**Published:** 2024-08-07

This is a correction to: Hao Wang, Junbo Yang, Yihong Cai, Yang Zhao, Macrophages suppress cardiac reprogramming of fibroblasts *in vivo* via IFN-mediated intercellular self-stimulating circuit, *Protein & Cell*, 2024; pwae013, https://doi.org/10.1093/procel/pwae013

In the originally published version of this paper, Figure 1 contained several typographical errors introduced during the editor’s production process, which have now been corrected:

- In Panel A, “*In Vivo* Dox induction” has been corrected to “*In Vitro* Dox induction”
- In Panel E the following labels have been corrected on the X axis:
  - o On the left hand side, “sh*Ifnar2#1*” has been corrected to “sh*Ifnar1#1*” and “sh*Ifnar2#2*” has been corrected to “sh*Ifnar1#2*”.
  - o On the right hand side, “sh*Ifnar2#1*” has been corrected to “sh*Ifnar1#1*” and “sh*Ifnar2#2*” has been corrected to “sh*Ifnar1#2*”

