# Supplementary material for: Macrophages suppress cardiac reprogramming of fibroblasts in vivo via IFN-mediated intercellular self-stimulating circuit
Source: Protein Cell. 2024 Mar 26;15(12):906–29. doi: 10.1093/procel/pwae013 (PMC11637486; doi:10.1093/procel/pwae013)
Supplement: pwae013_suppl_Supplementary_Material [file pwae013_suppl_supplementary_material.pdf]

Supplementary Materials

Supplementary Fig. Legends

Fig. S1: Gene set enrichment analysis (GSEA) of transcriptional profiles in MICFs from *in vivo* and *in vitro* samples

A: Heat map analysis of cardiomyocyte and cardiac fibroblast related genes of *in vivo* and *in vitro* samples. **B** and **C**: GSEA analysis using hallmark gene sets for the differences pathways in *in vivo* samples versus *in vitro* samples at 1 week (B) and 2 weeks (C). **D** and **E**: Scatter plot of differentially expressed genes in MICFs from *in vivo* and *in vitro* samples at 1 week (D) or 2 weeks (E). FPKM, fragments per kilobase of transcript per million mapped reads.

Fig. S2: Silencing *Ifnar1* or *Ifnar2* enhances cardiac reprogramming efficiency in MICFs

A: qPCR analysis validated knockdown efficiency of shRNAs targeting *Ifnar1* or *Ifnar2*. n=3. **B** and **C**: Representative IF images for α-actinin (red) on MICFs infected with MGT or MGTMyoS and sh*Ifnar1/2* or sh*NT* (B), with quantification of the absolute number (C). n=4. **D** and **E**: Representative whole-well images for α-actinin (green) and cTnI (red) on MICFs infected with MGT and sh*Ifnar1/2* or sh*NT* after 4 weeks. **F**: Representative flow plots for α-actinin$^+$ and cTnI$^+$ cells reprogrammed from MICFs 28 days after lentiviruses infection.

All data are presented as the means ± SD. The one-way ANOVA (A) or Two-way ANOVA (C) was used to determine the significance of differences between two groups. NS, not significant, *p < 0.05, **p < 0.01, *** p < 0.001, Scale bars, 100 μm.

Fig. S3: Efficient cardiac reprogramming by silencing *Ifnar2 in vivo*

A and **B**: Representative IF images for MYL2 (red), cTnI (grey), and GFP (green) after transplantation of MICFs expressing MGT and sh*Ifnar2* into MI hearts 4 weeks (A), with quantification of the percentage in (B). n=4. **C**: Schematic diagram showing the genetic fate mapping method to trace the lineage of resident MICFs using *Col1a2*^{CreER}/*R26*-tdTomato mice. **D** and **E**: Representative IF images for tdTomato (red) and GFP (green) after injection of LV-GFP & LV-rtTA into *Col1a2*^{CreER}/*R26*-tdTomato MI hearts 4 weeks (E), with quantification of the percentage GFP to tdTomatoin in (D). n=4. **F** and **G**: Representative IF images for α-actinin (green) and cTnI (red) on MICFs infected with MGT-HA and sh*Ifnar2/NT*-rtTA or sh*Ifnar2/NT* **+** rtTA (F), with quantification of the absolute number in (G). n=3. **H**: Representative IF images for tdTomato (red) and α-actinin (green) in **adjacent transverse** sections after injection of LV-MGT-HA and LV-shRNA-rtTA into *Col1a2*^{CreER}/*R26*-tdTomato MI hearts for 4 weeks. **I**: Z stack images for HA tdTomato and cTnI (left panel) or tdTomato and α-actinin (right panel) positive iCMs. n=4. **J**: Schematic diagram shows the genetic fate mapping method to trace the lineage of resident CFs and fusion analyses using *Tcf21*^{CreER}/mTmG mice. mGFP+ cardiomyocytes indicate cardiac reprogramming, whereas mGFP+ mtdTomato+ cardiomyocytes indicate cell fusion between CFs and cardiomyocytes. **K** and **L**: Representative IF images on mTdtomato (red), mGFP (green) and cTnI (grey) after injection of LV-MGT-HA and LV-shRNA-rtTA into MI hearts after 4 weeks (K), with

quantification of the percentage in (L). n=4. **M** and **N**: Representative IF images on mTdtomato (red), mGFP (green) and α-actinin (grey) in **adjacent transverse** sections after injection of LV-MGT-HA and LV-shRNA-rtTA into MI hearts after 4 weeks (M), with quantification of the CM markers percentage (N). n=4. **O**: Representative IF images on iCMs exhibiting well sarcomeric organization after injection of LV-MGT-HA and LV-sh*Ifnar2*-rtTA into MI hearts after 4 weeks. mTdtomato (red), mGFP (green) and α-actinin (grey).

All data are presented as the means ± SD. The unpaired t-test or Two-way ANOVA (G) was used to determine the significance of differences between two groups. NS, not significant, *p < 0.05, **p < 0.01, *** p < 0.001, Scale bars, 100 μm.

Fig. S4: Segmental analysis of indicated mice strains

A: Quantification of the longitudinal peak strains as from base to middle of anterior and posterior for the mice injected with GFP+sh*NT*, GFP+sh*Ifnar2*, MGT+sh*NT* or MGT+sh*Ifnar2* lentiviruses at day 28 (A) after MI, results were presented as violin plots. n=14-15.

All data are presented as the means ± SD. The Two-way ANOVA was used to determine the significance of differences between two groups. NS, not significant, *p < 0.05, **p < 0.01, *** p < 0.001.

Fig. S5: Macrophages secrete IFN-β in the injured heart

A: Sc-RNA seq analysis of *Ifnb1* and *Ifna2* expression in injured hearts. **B:** A table summarizing the expression of type 1 interferons in the injured heart. **C**: RT-qPCR analyses for *Ifnb1* expression in hearts at 3, 5-, 14-, 21- and 28-days post MI. n=3. **D**: Public Sc-RNA seq analysis of *Ifnb1* expression for each cell types.CM: Cardiomyocytes, CF: Cardiac fibroblast; EC: Endothelial cells; Mp: Macrophage; GN: granulocyte; T: T cell. **E** and **F**: Representative IF images for CD31, CD68, CD8, Vimentini and cTnI on MICFs (E), with the quantification of the percentage in (F). n=3. **G**: Representative IF images for CD68 on MICFs with depleting macrophages or not (left panel), with the quantification of the percentage in (right panel) n=3. **H**: Representative IF images for cTnI(red) and α-actinin (green) on MGT+sh*Ifnar2*/*NT* transduced MICFs with depleting macrophages or not (left panel), with the quantification of the percentage in (right panel). n=4.

All data are presented as the means ± SD. The unpaired t-test (G) or Two-way ANOVA (H) was used to determine the significance of differences between two groups. NS, not significant, *p < 0.05, **p < 0.01, *** p < 0.001, Scale bars, 100 μm.

Fig. S6: Macrophages secrete IFN-β suppresses cardiac reprograming

A: FACS identification of CD11b$^+$ F4/80$^+$ BMDM cells (left panel) and bright filed image of BMDM (right panel). **B**: Culture medium IFN-β concentration in WT or *Ifnb1*-KO BMDMs with the treatment of dsDNA for 24h. **C** and **D**: Representative IF images for cTnI(red) and α-actinin (green) on MGT+sh*Ifnar2*/*NT* transduced purified MICFs treated with KO-conditioned medium, conditioned medium or basal medium for 4 weeks (D),

with the quantification of the absolute number (C). n=4. **E**: Representative IF images for α-actinin (red), cTnI (grey), and GFP (green) on transplantation of MGT-transduced MICFs treated with CL2MDP into MI hearts 4 weeks, with CL2MDP injected time point at MI-1, MI-3. Red arrow indicates α-actinin^{+}cTnI^{-} iCMs. **F:** Quantification of α-actinin^{+} or cTnI^{+} iCMs. n=4. **G:** Survival rate of mice injected with PBS or CL2MDP at MI D1, 3, 5. n=6. **H** and **I**: Representative IF images for cTnI(red) and α-actinin (green) on MICFs infected with MGT and treated with IFNAR-neutralizing antibody or sh*Ifnar2* (H), with quantification of the absolute number and the time window of IFNAR-neutralizing antibody (I). n=3.

All data are presented as the means ± SD. The Two-way ANOVA (C), One-way ANOVA (F, I) or unpaired t-test (B) was used to determine the significance of differences between two groups. NS, not significant, *p < 0.05, **p < 0.01, *** p < 0.001, Scale bars, 100 μm.

Fig. S7: Efficient cardiac reprogramming with KD *Ifnar1/2* was not due to enhanced iCM proliferation.

A: Cell numbers difference between sh*NT* and sh*Ifnar1/2*-infected MICFs after MGT transduction. Cell numbers were counted by nuclear staining at 5- 7- and 14- days after induction. n = 3. **B**: mRNA expression of proliferation-related marker genes in sh*NT* or sh*Ifnar1/2* group in MICFs at 5-,10-,15- days after MGT transduction. n = 3. **C**: Representative IF images for Ki-67 (green) and cTnI (red) on MICFs infected with MGT and sh*Ifnar1/2* or sh*NT* at day5, 10 and 15 after transduction. n=3.**D**: Schematic diagram of EdU treatment during cardiac reprogramming (upper panel), representative IF images for Edu (green) and cTnI (red) on MICFs infected with MGT and sh*Ifnar1/2* or sh*NT* at 4 weeks after transduction (lower panel). n=3. **E**: Representative IF images for MGT-HA (red) on MICFs infected with MGT-HA and sh*Ifnar2* or sh*NT* at day7 (left panel), with the quantification of percentage in (right panel). n=3. **F**: Representative IF images for STAT1-GFP (green), p-STAT1(grey) and GATA4 (red) on purified MICFs with the treatment of 100 IU/ml IFN-β for 1 hour. **G:** Quantification of the percentage of nuclear STAT1-GFP^{+} & p-STAT1^{+} cells/STAT1-GFP^{+} cells. n=8. **H**: Density plots showing signals of Cut-tag at GATA4 binding sites between control, STAT-i and sh*Ifnar2* group. **I**: CUT&Tag peak heatmap showing GATA4 binding loci enriched in control group but down-regulated with the treatment of sh*Ifnar2* or STAT-i. **J**: TRANSFAC and JASPAR PWMs analysis of the regions that enriched in infected MICFs with the treatment of sh*NT*+DMSO compared to sh*Ifnar2* group (left panel) or STAT-i group (right panel). **K**: GSEA enrichment plots show that STAT1 targets are enriched in control group but down-regulated in sh*Ifnar2* or STAT1-I group. **L**: IGV tracks showing GATA4 CUT&Tag at *Oas2* and *Oas3* gene locus between control, STAT-i and sh*Ifnar2* group. **M**: Searching for Motif of STAT1::STAT2 in the control group.

All data are presented as the means ± SD. The One-way ANOVA (A,B) or unpaired t-test (E) was used to determine the significance of differences between two groups. NS, not significant, *p < 0.05, **p < 0.01, *** p < 0.001, Scale bars, 100 μm (F=10 μm).

Fig. S8: The expression of *Ifnb1* after silencing of *Ifnar2* in MI hearts

A: qPCR analysis validated knockdown efficiency of shRNAs targeting *Ccl2/7/12* compared to sh***NT***. n=3. **B**: Schematic diagram of dual luciferase experiments. **C**: STAT1

binding motifs from JASPAR database. **D**: Experimental workflow of RNA extraction from the isolated tissue of infarcted area (left panel) and LV-sh*Ifnar2*-mScarlet-H2B expression in the C56BL/6 mice infarct hearts (right panel). **E**: qPCR analysis of the *Ifnb1* expression from the infarcted area injected with LV- sh*Ifnar2*-mScarlet-H2B or sh*NT*-mScarlet-H2B at 7-, 14- days post MI. n=3. **F**: Representative IF images for CD68 (green) and CD3 (red) on *in vivo* knockdown experiment. n=3. **G**: Quantification of CD68 macrophages and CD3 T cells from *in vivo* knockdown experiment. **H**: qPCR analysis of the *Ifnb1* expression from the infarcted area administration of TMM/vehicle at 7- days post MI or sham.

All data are presented as the means ± SD. The unpaired t-test (A, E) or One-way ANOVA (G, H) was used to determine the significance of differences between two groups. NS, not significant, *p < 0.05, **p < 0.01, *** p < 0.001, Scale bars, 100 μm (D:1000 μm).

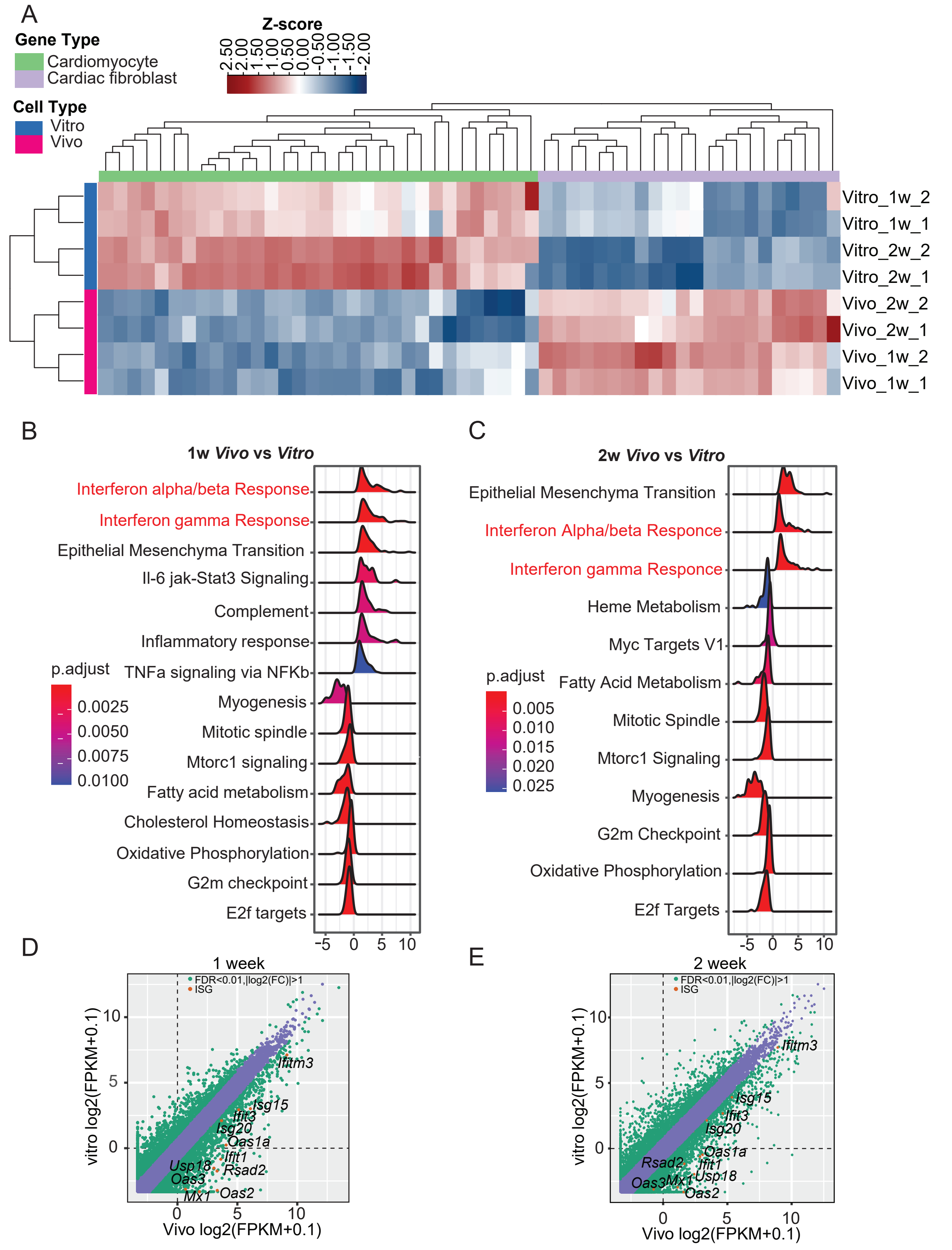

A
Gene Type
Cardiomyocyte
Cardiac fibroblast
Cell Type
Vitro
Vivo
Z-score
2.50
2.00
1.50
1.00
0.50
0.00
-0.50
-1.00
-1.50
-2.00
Vitro_1w_2
Vitro_1w_1
Vitro_2w_2
Vitro_2w_1
Vivo_2w_2
Vivo_2w_1
Vivo_1w_2
Vivo_1w_1
B
1w Vivo vs Vitro
Interferon alpha/beta Response
Interferon gamma Response
Epithelial Mesenchyma Transition
Il-6 jak-Stat3 Signaling
Complement
Inflammatory response
TNFa signaling via NFKb
Myogenesis
Mitotic spindle
Mtorc1 signaling
Fatty acid metabolism
Cholesterol Homeostasis
Oxidative Phosphorylation
G2m checkpoint
E2f targets
p.adjust
0.0025
0.0050
0.0075
0.0100
-5
0
5
10
C
2w Vivo vs Vitro
Epithelial Mesenchyma Transition
Interferon Alpha/beta Responce
Interferon gamma Responce
Heme Metabolism
Myc Targets V1
Fatty Acid Metabolism
Mitotic Spindle
Mtorc1 Signaling
Myogenesis
G2m Checkpoint
Oxidative Phosphorylation
E2f Targets
p.adjust
0.005
0.010
0.015
0.020
0.025
-5
0
5
10
D
1 week
FDR<0.01,|log2(FC)|>1
ISG
Ifitm3
Isg15
Ifit3
Isg20
Oas1a
Ifit1
Usp18
Rsad2
Oas3
Mx1
Oas2
vitro log2(FPKM+0.1)
Vivo log2(FPKM+0.1)
0
5
10
E
2 week
FDR<0.01,|log2(FC)|>1
ISG
Ifitm3
Isg15
Ifit3
Isg20
Oas1a
Rsad2
Ifit1
Oas3
Mx1
Usp18
Oas2
vitro log2(FPKM+0.1)
Vivo log2(FPKM+0.1)
0
5
10

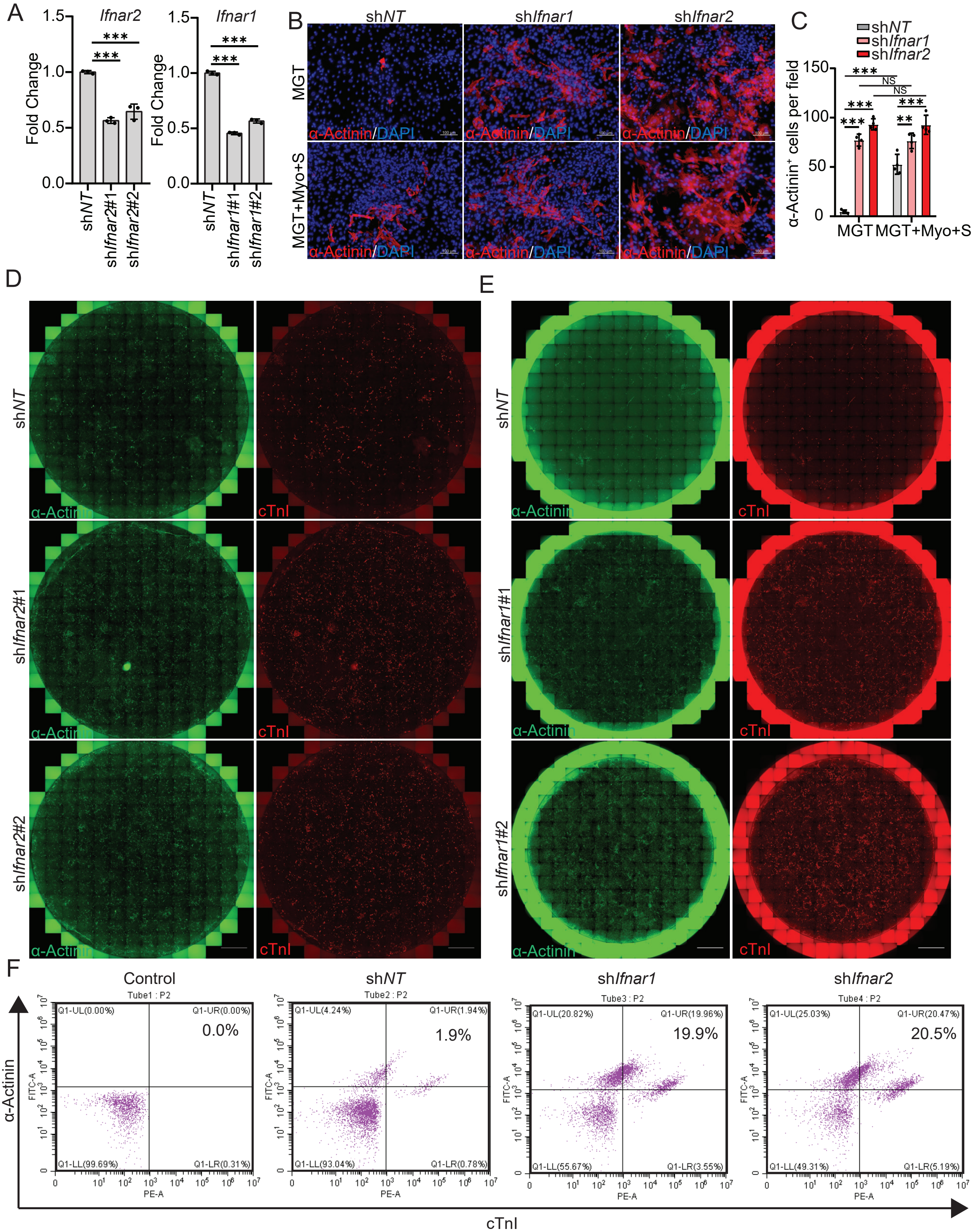

A
Ifnar2
Ifnar1
Fold Change
1.5
1.0
0.5
0.0
***
shNT
shIfnar2#1
shIfnar2#2
shIfnar1#1
shIfnar1#2
B
shNT
shIfnar1
shIfnar2
MGT
MGT+Myo+S
α-Actinin/DAPI
C
shNT
shIfnar1
shIfnar2
α-Actinin+ cells per field
150
100
50
0
NS
**
MGT
MGT+Myo+S
D
E
α-Actinin
cTnI
F
Control
Tube1 : P2
Q1-UL(0.00%)
Q1-UR(0.00%)
0.0%
Q1-LL(99.69%)
Q1-LR(0.31%)
shNT
Tube2 : P2
Q1-UL(4.24%)
Q1-UR(1.94%)
1.9%
Q1-LL(93.04%)
Q1-LR(0.78%)
shIfnar1
Tube3 : P2
Q1-UL(20.82%)
Q1-UR(19.96%)
19.9%
Q1-LL(55.67%)
Q1-LR(3.55%)
shIfnar2
Tube4 : P2
Q1-UL(25.03%)
Q1-UR(20.47%)
20.5%
Q1-LL(49.31%)
Q1-LR(5.19%)
FITC-A
PE-A
α-Actinin
cTnI

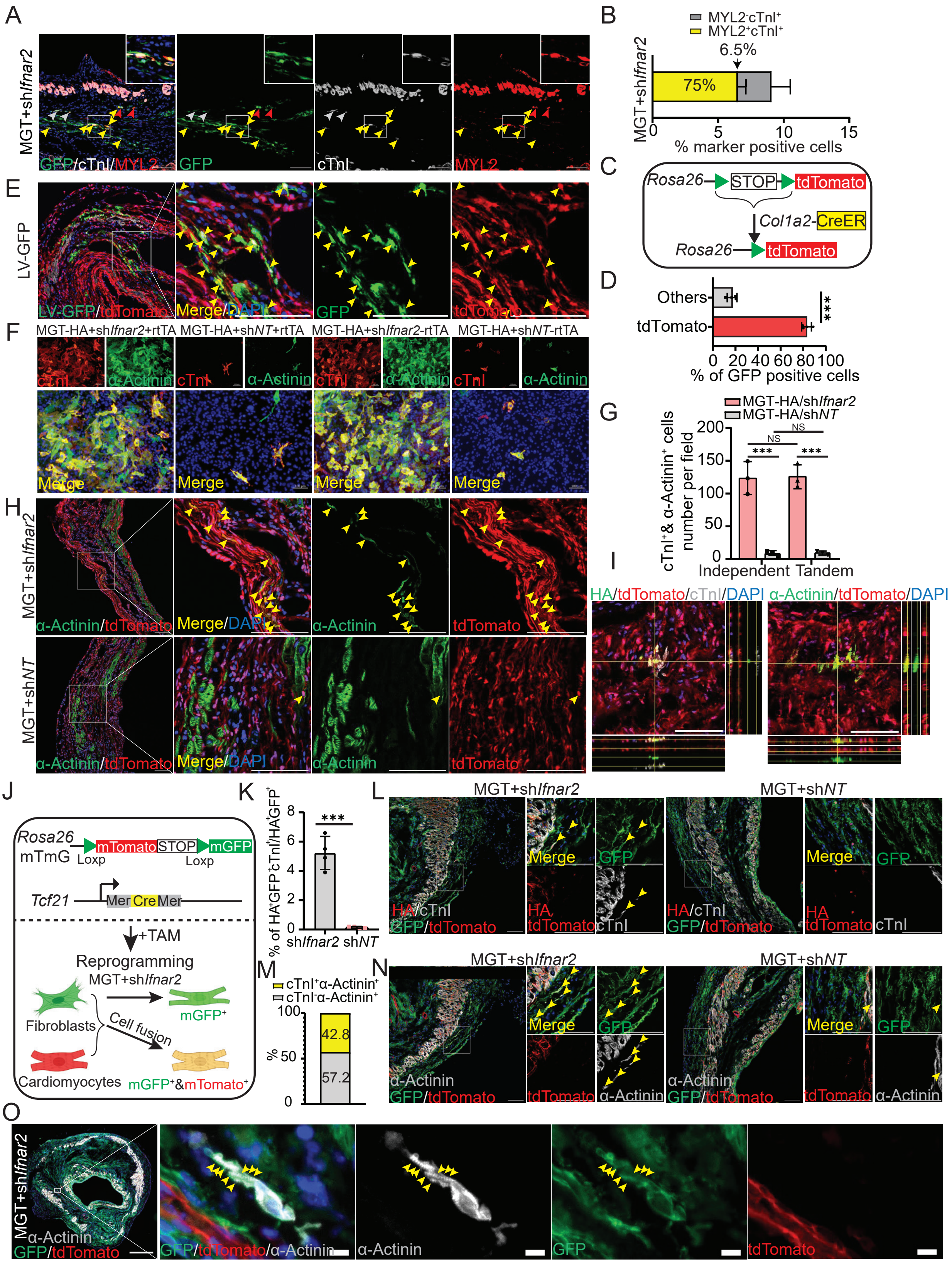

A
MGT+shIfnar2
GFP/cTnI/MYL2
GFP
cTnI
MYL2
B
MYL2⁻cTnI⁺
MYL2⁺cTnI⁺
6.5%
75%
MGT+shIfnar2
0
5
10
15
% marker positive cells
C
Rosa26
STOP
tdTomato
Col1a2-CreER
Rosa26
tdTomato
D
Others
tdTomato
***
0
20
40
60
80
100
% of GFP positive cells
E
LV-GFP
LV-GFP/tdTomato
Merge/DAPI
GFP
tdTomato
F
MGT-HA+shIfnar2+rtTA
MGT-HA+shNT+rtTA
MGT-HA+shIfnar2-rtTA
MGT-HA+shNT-rtTA
cTnI
α-Actinin
Merge
G
MGT-HA/shIfnar2
MGT-HA/shNT
cTnI⁺& α-Actinin⁺ cells number per field
200
150
100
50
0
NS
NS
***
***
Independent
Tandem
H
MGT+shIfnar2
MGT+shNT
α-Actinin/tdTomato
Merge/DAPI
α-Actinin
tdTomato
I
HA/tdTomato/cTnI/DAPI
α-Actinin/tdTomato/DAPI
J
Rosa26
mTmG
mTomato
STOP
mGFP
Loxp
Loxp
Tcf21
Mer
Cre
Mer
+TAM
Reprogramming
MGT+shIfnar2
Fibroblasts
mGFP⁺
Cell fusion
Cardiomyocytes
mGFP⁺&mTomato⁺
K
% of HA⁺GFP⁺cTnI⁺/HA⁺GFP⁺
8
6
4
2
0
***
shIfnar2
shNT
L
MGT+shIfnar2
MGT+shNT
Merge
GFP
HA/cTnI
GFP/tdTomato
HA
tdTomato
cTnI
M
cTnI⁺α-Actinin⁺
cTnI⁻α-Actinin⁺
100
50
0
%
42.8
57.2
N
MGT+shIfnar2
MGT+shNT
Merge
GFP
α-Actinin
GFP/tdTomato
tdTomato
α-Actinin
O
MGT+shIfnar2
α-Actinin
GFP/tdTomato
GFP/tdTomato/α-Actinin
α-Actinin
GFP
tdTomato

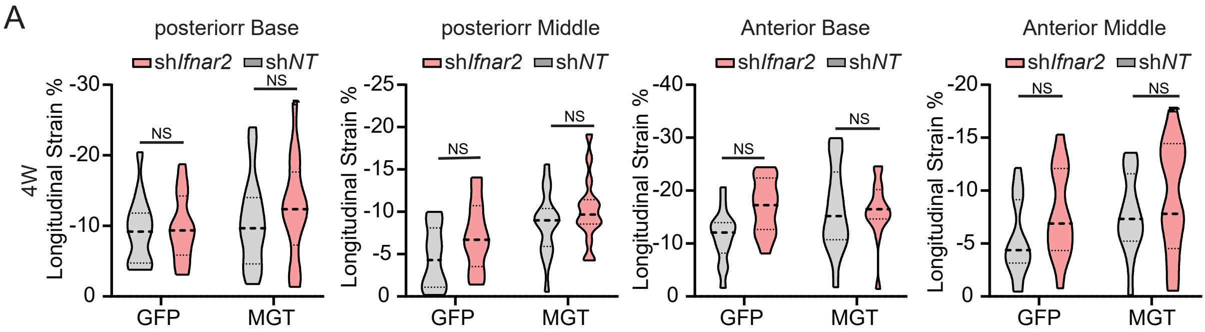
A
posteriorr Base
posteriorr Middle
Anterior Base
Anterior Middle
shIfnar2
shNT
4W
Longitudinal Strain %
NS
GFP
MGT

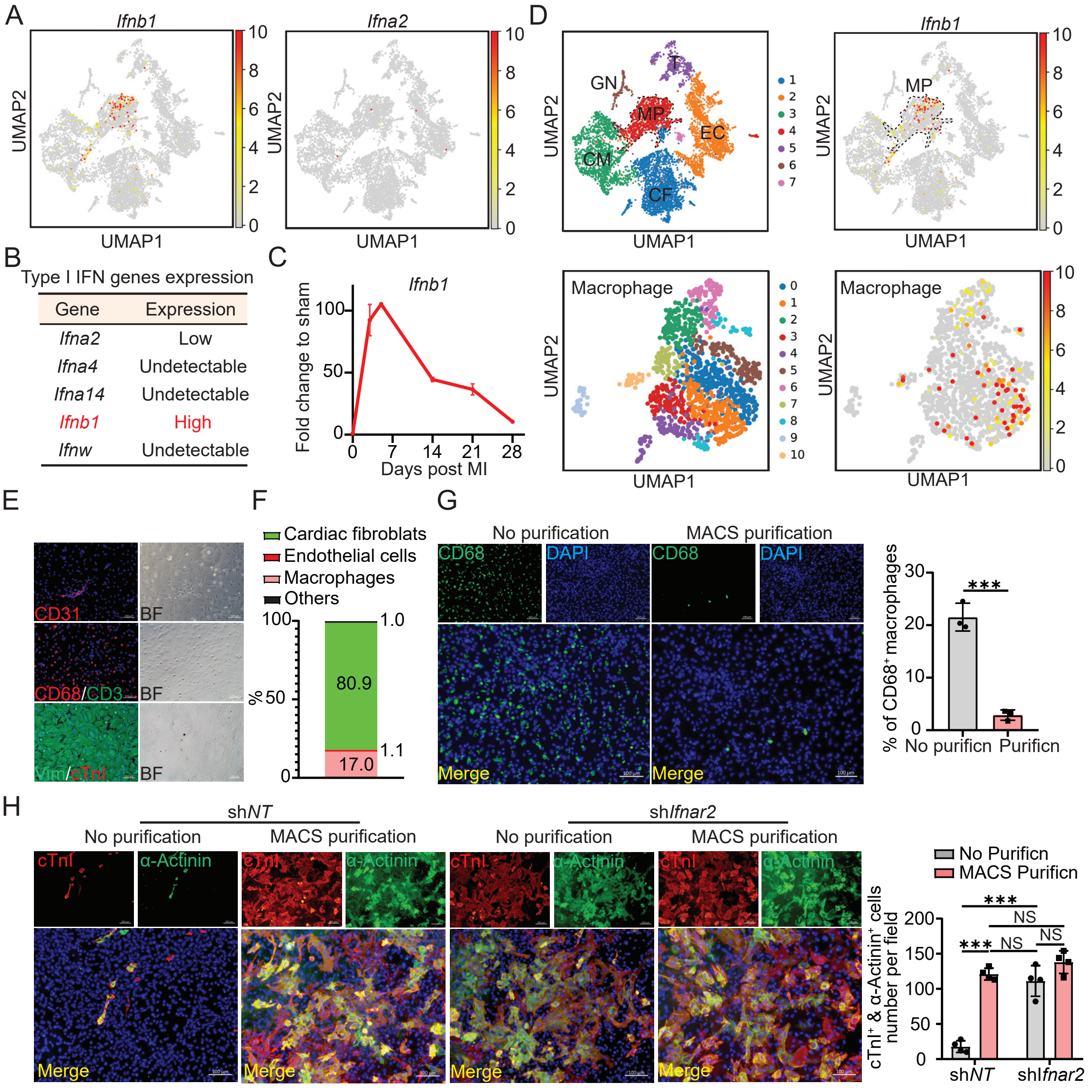
A
Ifnb1
Ifna2
UMAP1
UMAP2
B
Type I IFN genes expression
Gene Expression
Ifna2 Low
Ifna4 Undetectable
Ifna14 Undetectable
Ifnb1 High
Ifnw Undetectable
C
Ifnb1
Fold change to sham
Days post MI
D
GN
T
MP
EC
CM
CF
Macrophage
E
CD31
BF
CD68/CD3
Vim/cTnI
F
Cardiac fibroblats
Endothelial cells
Macrophages
Others
%
1.0
80.9
1.1
17.0
G
No purification
MACS purification
CD68
DAPI
Merge
% of CD68+ macrophages
***
No purificn
Purificn
H
shNT
shIfnar2
No purification
MACS purification
cTnI
α-Actinin
Merge
No Purificn
MACS Purificn
cTnI+ & α-Actinin+ cells number per field
***
NS

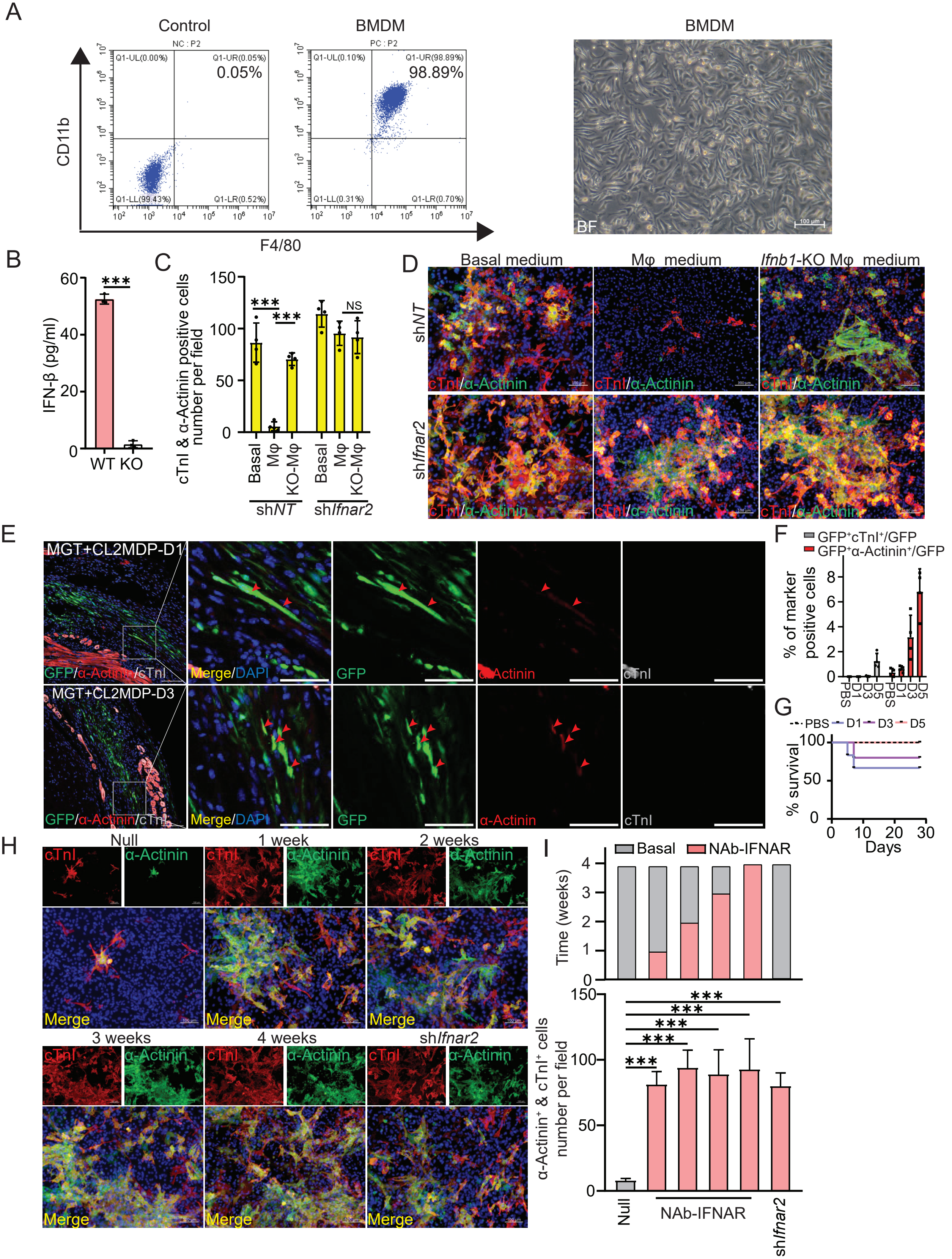
A
Control
BMDM
BMDM
CD11b
F4/80
0.05%
98.89%
BF
B
IFN-β (pg/ml)
WT KO
C
cTnI & α-Actinin positive cells number per field
Basal
Mφ
KO-Mφ
shNT
shIfnar2
NS
D
Basal medium
Mφ medium
Ifnb1-KO Mφ medium
shNT
shIfnar2
cTnI/α-Actinin
E
MGT+CL2MDP-D1
MGT+CL2MDP-D3
GFP/α-Actinin/cTnI
Merge/DAPI
GFP
α-Actinin
cTnI
F
GFP+cTnI+/GFP
GFP+α-Actinin+/GFP
% of marker positive cells
PBS D1 D3 D5 PBS D1 D3 D5
G
PBS D1 D3 D5
% survival
Days
H
Null
1 week
2 weeks
3 weeks
4 weeks
shIfnar2
cTnI
α-Actinin
Merge
I
Basal
NAb-IFNAR
Time (weeks)
α-Actinin+ & cTnI+ cells number per field
Null
NAb-IFNAR
shIfnar2

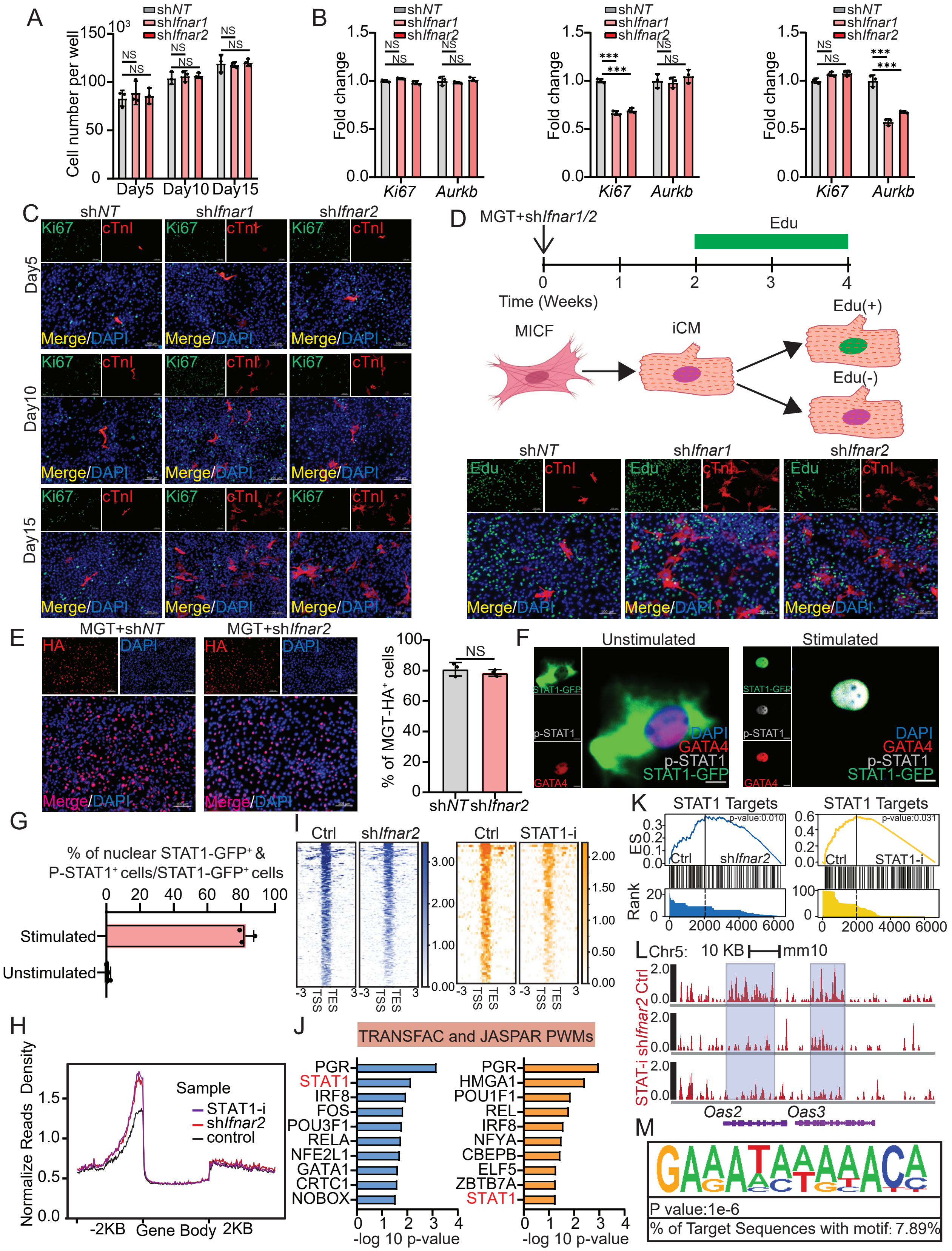

A
B
C
D
E
F
G
H
I
J
K
L
M
shNT
shIfnar1
shIfnar2
Cell number per well
Day5
Day10
Day15
Fold change
Ki67
Aurkb
cTnI
Merge/DAPI
MGT+shIfnar1/2
Edu
Time (Weeks)
MICF
iCM
Edu(+)
Edu(-)
MGT+shNT
MGT+shIfnar2
HA
DAPI
% of MGT-HA⁺ cells
Unstimulated
Stimulated
STAT1-GFP
p-STAT1
GATA4
% of nuclear STAT1-GFP⁺ & P-STAT1⁺ cells/STAT1-GFP⁺ cells
Ctrl
STAT1-i
TSS
TES
Normalize Reads Density
Sample
control
-2KB
Gene Body
2KB
TRANSFAC and JASPAR PWMs
PGR
STAT1
IRF8
FOS
POU3F1
RELA
NFE2L1
GATA1
CRTC1
NOBOX
HMGA1
POU1F1
REL
NFYA
CBEPB
ELF5
ZBTB7A
-log 10 p-value
STAT1 Targets
p-value:0.010
p-value:0.031
ES
Rank
Chr5:
10 KB
mm10
Oas2
Oas3
P value:1e-6
% of Target Sequences with motif: 7.89%

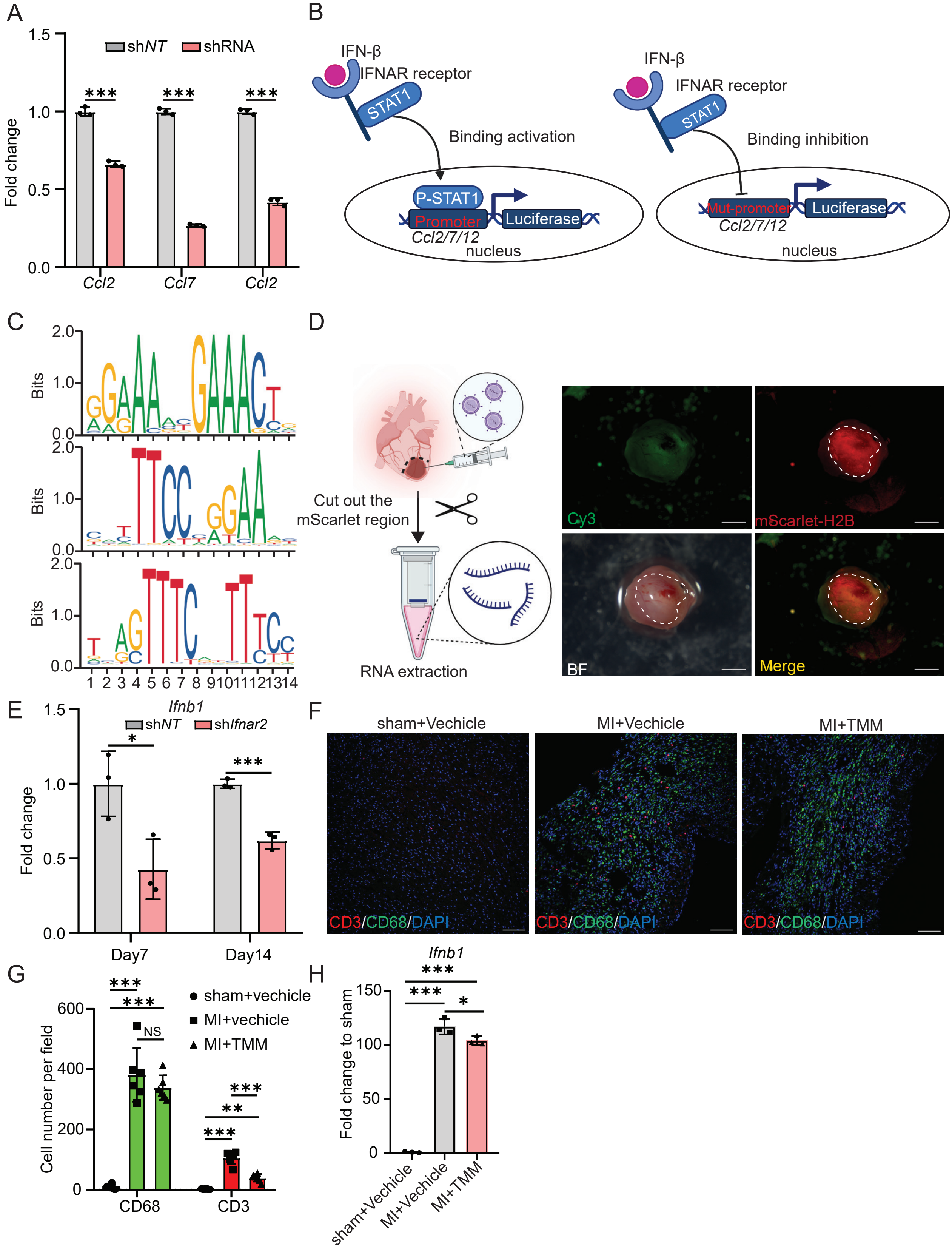
A
shNT
shRNA
Fold change
1.5
1.0
0.5
0.0
***
***
***
Ccl2
Ccl7
Ccl2
B
IFN-β
IFNAR receptor
STAT1
Binding activation
P-STAT1
Promoter
Luciferase
Ccl2/7/12
nucleus
IFN-β
IFNAR receptor
STAT1
Binding inhibition
Mut-promoter
Luciferase
Ccl2/7/12
nucleus
C
Bits
2.0
1.0
0.0
1 2 3 4 5 6 7 8 9 10 11 12 13 14
D
Cut out the mScarlet region
RNA extraction
Cy3
mScarlet-H2B
BF
Merge
E
Ifnb1
shNT
shIfnar2
Fold change
1.5
1.0
0.5
0.0
*
***
Day7
Day14
F
sham+Vechicle
MI+Vechicle
MI+TMM
CD3/CD68/DAPI
CD3/CD68/DAPI
CD3/CD68/DAPI
G
sham+vechicle
MI+vechicle
MI+TMM
Cell number per field
600
400
200
0
***
***
NS
***
**
***
CD68
CD3
H
Ifnb1
Fold change to sham
150
100
50
0
***
***
*
sham+Vechicle
MI+Vechicle
MI+TMM

Table S1. Related to STAR Methods.

Sequence of shRNA and sgRNA

Gene	Forward Sequence& Reverse Sequence	Target sequence
Ifnar1-shRNA-1	CCGGGGGAAATCACACATCCTTTCTCGAGAAAGGATGTGTGATTTCCCTTTTTG<br>AATTCAAAAAGGGAAATCACACATCCTTTCTCGAGAAAGGATGTGTGATTTCCC	GGGAAATCACACATCCTTT
Ifnar1-shRNA-2	CCGGGCAAAGACATGTAAGCAAACTCGAGTTTGCTTACATGTCTTTGCTTTTTG<br>AATTCAAAAAGCAAAGACATGTAAGCAAACTCGAGTTTGCTTACATGTCTTTGC	GCAAAGACATGTAAGCAAA
Ifnar2-shRNA-1	CCGGGTCTTGAACTTCCGCCACTTTCTCGAGAAAGTGGCGGAAGTTCAAGACTTTTTG<br>AATTCAAAAAGTCTTGAACTTCCGCCACTTTCTCGAGAAAGTGGCGGAAGTTCAAGAC	GTCTTGAACTTCCGCCACTTT
Ifnar2-shRNA-2	CCGGGGGAGAGAAAGGGAAAGAACTCGAGTTCTTTCCCTTTCTCTCCCTTTTTG<br>AATTCAAAAAGGGAGAGAAAGGGAAAGAACTCGAGTTCTTTCCCTTTCTCTCCC	GGGAGAGAAAGGGAAAGAA
Ccl2-sh*RNA*	CCGGGCAAGATGATCCCAATGAGTACTCGAGTACTCATTGGGATCATCTTGCTTTTTG	GCAAGATGATCCCAATGAGTA
Ccl7-sh*RNA*	CCGGCGAGGAGGCTATAGCATACTTCTCGAGAAGTATGCTATAGCCTCCTCGTTTTTG	CGAGGAGGCTATAGCATACTT
Ccl12-sh*RNA*	CCGGGCTGTGATCTTCAGGACCATACTCGAGTATGGTCCTGAAGATCACAGCTTTTTG	GCTGTGATCTTCAGGACCATA

sh*NT*	CCGGCCTAAGGTTAAGTCGCCCTCGCTCGAGCGAGGGCGACTTAACCTTAGGTTTTTG<br>AATTCAAAAACCTAAGGTTAAGTCGCCCTCGCTCGAGCGAGGGCGACTTAACCTTAGG	CCTAAGGTTAAGTCGCCCTCG
sg*Ifnb1*-1	ATCTTGAAGTCCGCCCTGTAGG	ATCTTGAAGTCCGCCCTGT
sg*Ifnb1*-2	GAAGTACAACAGCTACGCCTGG	GAAGTACAACAGCTACGCC

Table S2. Sequences for Primer.   Related to STAR Methods

1.Primer sequence of qPCR

**Gene**	**Forward**	**Reverse**
Myh6	GCCCAGTACCTCCGAAAGTC	GCCTTAACATACTCCTCCTTGTC
Actc1	CTGGATTCTGGCGATGGTGTA	CGGACAATTTCACGTTCAGCA
Tnnt2	CAGAGGAGGCCAACGTAGAAG	CTCCATCGGGGATCTTGGGT
Ryr2	ACGGCGACCATCCACAAAG	AAAGTCTGTTGCCAAATCCTTCT
Ccl2	TTAAAAACCTGGATCGGAACCAA	GCATTAGCTTCAGATTTACGGGT
Ccl7	GCTGCTTTCAGCATCCAAGTG	CCAGGGACACCGACTACTG
Ccl12	ATTTCCACACTTCTATGCCTCCT	ATCCAGTATGGTCCTGAAGATCA
Ifnar1	AGCCACGGAGAGTCAATGG	GCTCTGACACGAAACTGTGTTTT
Ifnar2	CTTCGTGTTTGGTAGTGATGGT	GGGGATGATTTCCAGCCGA
Ifnb1	CAGCTCCAAGAAAGGACGAAC	GGCAGTGTAACTCTTCTGCAT
Ki67	ATCATTGACCGCTCCTTTAGGT	GCTCGCCTTGATGGTTCCT
Aurkb	CAGAAGGAGAACGCCTACCC	GAGAGCAAGCGCAGATGTC
Gapdh	AGGTCGGTGTGAACGGATTTG	TGTAGACCATGTAGTTGAGGTCA

2. Mouse Genotyping Primer Sequences

**Mouse line**	**Forward**	**Reverse**

Col1a2-CreER	AATAAGCCATCTCGCCTGCCGTTC	GGATAGTTTTTACTGCCAGACCGC (Mut)<br>TCGTGTCAGCCTTGGTCAACTCTC (WT)
Tcf21-CreER	TCAATGGGCGGGGGTCGTT	TCTTGCGGGGTGGGATAGGGAG
Rosa-mTmG	CTCTGCTGCCTCCTGGCTTCT	CGAGGCGGATCACAAGCAATA (WT)<br>TCAATGGGCGGGGGTCGTT (Mut)
Rosa-tdTomato	AAGGGAGCTGCAGTGGAGTA(WT)<br>CTGTTCCTGTACGGCATGG(Mut)	CCGAAAATCTGTGGGAAGTC (WT)<br>GGCATTAAAGCAGCGTATCC (Mut)
